# Internal guide RNA interactions interfere with Cas9-mediated cleavage

**DOI:** 10.1038/ncomms11750

**Published:** 2016-06-10

**Authors:** Summer B. Thyme, Laila Akhmetova, Tessa G. Montague, Eivind Valen, Alexander F. Schier

**Affiliations:** 1 Department of Molecular and Cellular Biology, Harvard University, 16 Divinity Avenue, BIOL 1020, Cambridge, Massachusetts 02138, USA; 2 Computational Biology Unit, Department of Informatics, University of Bergen, 5020 Bergen, Norway; 3 Sars Centre for Marine Molecular Biology, University of Bergen, Thormøhlensgaten 55, 5008 Bergen, Norway; 4 Department of Molecular and Cellular Biology, Harvard University, Cambridge, Massachusetts 02138, USA; 5 Center for Brain Science, Harvard University, Cambridge, Massachusetts 02138, USA; 6 Broad Institute of MIT and Harvard, Cambridge, Massachusetts 02142, USA; 7 Harvard Stem Cell Institute, Cambridge, Massachusetts 02138, USA; 8 FAS Center for Systems Biology, Harvard University, Harvard, Massachusetts 02138, USA

## Abstract

The CRISPR/Cas system uses guide RNAs (gRNAs) to direct sequence-specific DNA cleavage. Not every gRNA elicits cleavage and the mechanisms that govern gRNA activity have not been resolved. Low activity could result from either failure to form a functional Cas9–gRNA complex or inability to recognize targets *in vivo*. Here we show that both phenomena influence Cas9 activity by comparing mutagenesis rates in zebrafish embryos with *in vitro* cleavage assays. *In vivo*, our results suggest that genomic factors such as CTCF inhibit mutagenesis. Comparing near-identical gRNA sequences with different *in vitro* activities reveals that internal gRNA interactions reduce cleavage. Even though gRNAs containing these structures do not yield cleavage-competent complexes, they can compete with active gRNAs for binding to Cas9. These results reveal that both genomic context and internal gRNA interactions can interfere with Cas9-mediated cleavage and illuminate previously uncharacterized features of Cas9–gRNA complex formation.

## Abstract (web summary)

While the CRISPR-Cas9 system has revolutionised molecular biology, it is still a mystery why not every guide RNA elicits target DNA cleavage. Here the authors show that genomic context and internal gRNA interactions can inhibit cleavage.

## Graphical abstract

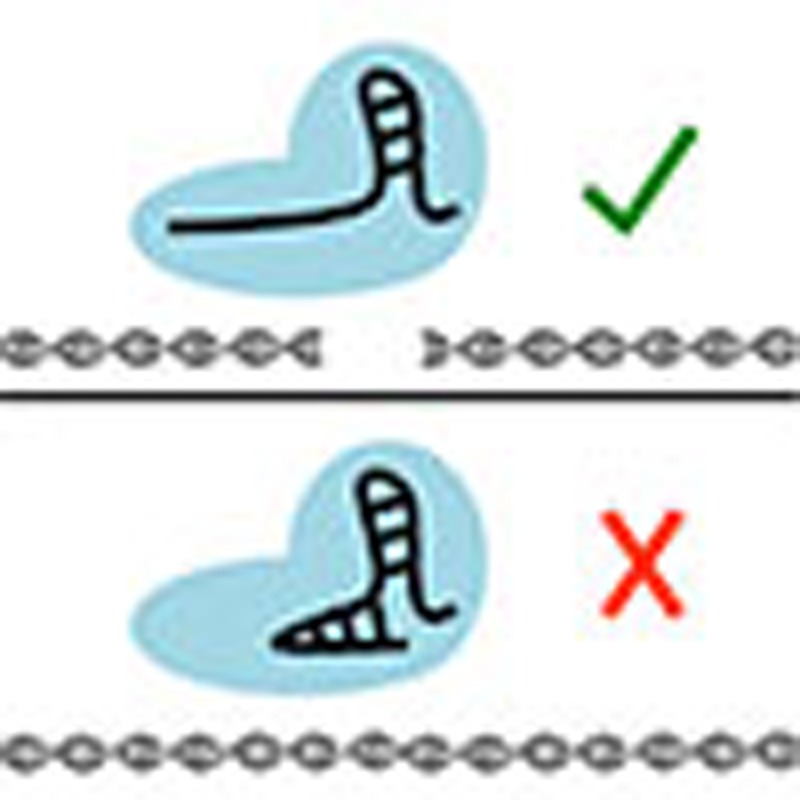

The CRISPR/Cas system is a revolutionary genome-editing technology^1^,^2^,^3^,^4^,^5^ in which the Cas9 protein binds to a guide RNA (gRNA) that directs sequence-specific cleavage via complementarity to a DNA target. The cleavage activities of thousands of Cas9 gRNAs have been tested and have revealed rules for high gRNA activity, including GC content of >50% and a preference for guanine adjacent to the PAM motif at position 20 (refs 6, 7, 8, 9, 10, 11). However, these rules are not sufficient to explain observed cutting rates *in vivo*. For example, zebrafish gRNAs that obey these rules do not always elicit high rates of mutagenesis^8^,^11^. Identifying the underlying reasons is of great importance, both to improve our understanding of the mechanisms that control gRNA–Cas9 activity and to develop better tools for Cas9-mediated genome editing.

There are two potential mechanisms that might contribute to poor gRNA performance: first, some gRNAs could be inherently poor at forming active Cas9–gRNA complexes, or second, they do form active complexes but their target sites could be refractory *in vivo*. By measuring both the *in vitro* cleavage and *in vivo* activity in zebrafish embryos, we find that there are gRNAs that fail for both reasons. Our data suggest that gRNAs that cleave well *in vitro* but not *in vivo*, are blocked by sequence-specific genomic factors and chromatin. By further dissection of the gRNAs that fail to form a functional Cas9 complex, we found that they contain internal gRNA interactions, providing experimental support for suggestions that gRNA secondary structure modulates Cas9 cleavage efficiency^9^,^10^,^12^. Although their fold prevents recognition activity, we found that these gRNAs can still bind to the Cas9 protein, competing effectively with active gRNAs. Strikingly, some inactive complexes can regain high levels of activity when internal gRNA interactions are disrupted. These results contribute to the groundwork for future structural studies on inactive Cas9–gRNA complexes and the mechanism of gRNA loading, and provide new rules for improving gRNA design methods.

## Results

### Short genomic regions can be refractory to Cas9 cleavage

**Figure 1:**
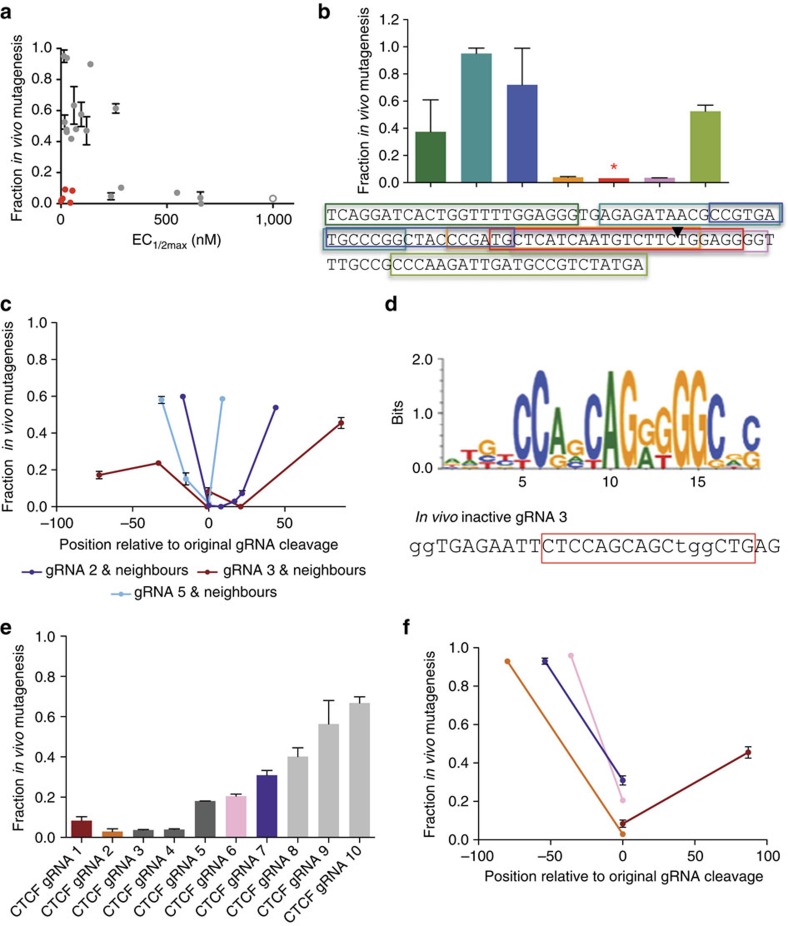
Genomic regions refractory to Cas9 cleavage are short and can be sequence dependent. (**a**) Mutagenesis in zebrafish embryos versus *in vitro* enzymatic cleavage for multiple gRNAs. Points in red represent five gRNAs with high enzymatic cleavage *in vitro* (low EC_1/2max_) that induce low mutagenesis. Examples covering a wide range of different gRNA activities are shown in grey. The open circle represents a gRNA with an EC_1/2max_ too high (>1,000 nM) to be accurately measured under tested conditions. (**b**) Cleavage activity and sequence locations, shown on a continuous region of the genome, of gRNAs surrounding and overlapping with one of the five gRNAs (*in vivo* inactive gRNA 1, marked with a star) highlighted in **a**. The cleavage location of the original gRNA is marked with a black triangle. The two gRNAs overlapping with the original gRNA display low mutagenesis, despite high *in vitro* activity (Supplementary Fig. 1). (**c**) Cleavage activity and relative location of neighbouring and overlapping gRNAs (Supplementary Table 1) compared with the original cleavage site for three *in vivo* inactive gRNAs. The relative position is the distance in nucleotides between the cleavage location of the original inactive gRNA and the neighbour. (**d**) Sequence logo representing CTCF-binding preferences in human cells^30^,^31^. The region of *in vivo* inactive gRNA 3 containing a putative CTCF site is boxed in red. The PAM sequence and first two bases of gRNA target are in lowercase. (**e**) Cleavage activity of gRNAs containing putative CTCF sequences (Supplementary Table 1), matching the sequence in inactive gRNA 3 shown in **d**. Three gRNAs (pale grey, 8–10) cleave with high efficiency (>0.4), three are low cleaving (0.2–0.3, 5–7) and four are completely inhibited (<0.1, 1–4). The colours of gRNAs 1, 2, 6 and 7 correspond to colours in **f**, where neighbouring gRNAs were tested. (**f**) Cleavage activity of gRNAs closely neighbouring four gRNAs with repressed cleavage and putative CTCF-binding sites. Line colour corresponds to bar colour shown in **e**. At least two independent assays were completed for each mutagenesis or *in vitro* cleavage experiment and error bars shown in all panels are s.e.m.

To determine *in vitro* cleavage rates, we used single turnover enzyme cleavage assays^1^,^13^, in which the concentration of Cas9 protein was varied in the presence of a lower amount of DNA substrate and an excess of gRNA. Activity was defined as the concentration of Cas9 that yielded half-maximal cleavage (EC_1/2max_), determined by quantifying fraction of substrate and product at multiple Cas9 concentrations. Comparing the results of this assay with cleavage in zebrafish embryos^8^, we identified gRNAs with high *in vitro* cleavage activity (low EC_1/2max_) but low *in vivo* mutagenesis rates (Fig. 1a). To determine whether these differences might be caused by target sites that are refractory *in vivo* to the Cas9 complex, we probed the cleavability of the region containing each of these five target sites by testing additional gRNAs that overlap with and surround the original gRNA binding sequence. We found that four of the five gRNAs (labelled *in vivo* inactive gRNAs 1–5) were located within short, sometimes fewer than 50-nucleotide, regions that were refractory to cleavage (Fig. 1b,c). For each of the five refractory regions we evaluated whether low gRNA activity might be sequence dependent or whether chromatin might play a role. We identified genomic sequences with partial sequence complementarity to *in vivo* inactive gRNAs 1 and 2, and found that some of these sites were also refractory to cleavage (Supplementary Fig. 2). For gRNA 3 we identified putative binding sites for CTCF, a protein involved in genome organization^14^. Target sites containing putative CTCF motifs (Fig. 1d) were often refractory to *in vivo* cleavage (Fig. 1e and Supplementary Table 1), whereas closely neighbouring sites were cleaved with very high efficiency (Fig. 1f). We found that the target sites of *in vivo* inactive gRNA 5 and *in vivo* inactive gRNA 4, the one gRNA for which we did not find clear borders of the refractory region, are both bound by modified histones^15^,^16^, whereas the other three are not (Supplementary Fig. 3). These results reveal that gRNAs that are active *in vitro* can be inactive at short genomic regions *in vivo*, and that many of these regions have identifiable characteristics that enable potential prediction and avoidance.

### Inactive gRNAs bind to Cas9 protein

**Figure 2:**
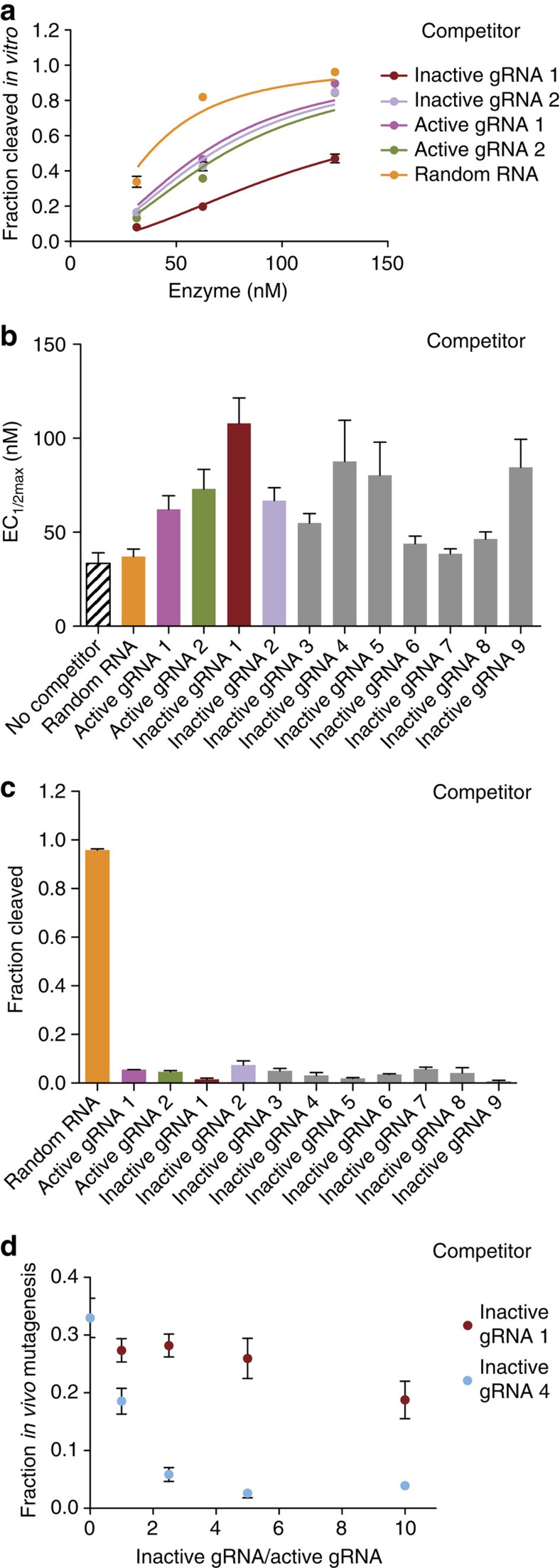
Inactive gRNAs bind to Cas9 protein. (**a**) Example target site cleavage traces for a highly active gRNA–Cas9 complex combined with an equivalent amount of competitor inactive gRNA (Supplementary Table 2), active gRNA (Supplementary Table 3) or control random RNA (Supplementary Methods). The inactive gRNAs elicit no cleavage at the enzyme concentrations tested. (**b**) EC_1/2max_ values for a highly active gRNA–Cas9 complex without a competitor (black striped bars) or combined with an equivalent amount of nine inactive gRNAs, two active gRNAs or control random RNA. (**c**) After prior incubation of 125 μM Cas9 protein with nine inactive gRNAs, two active gRNAs or control random RNA for 15 min, the cleavage for an active gRNA at its target was measured. (**d**) *In vivo* cleavage activity for a highly active gRNA (active gRNA 3, Supplementary Table 3) with Cas9 mRNA combined with a varying amount of each of two inactive gRNAs. At least two independent assays were completed for each mutagenesis or *in vitro* cleavage experiment and error bars shown in all panels are s.e.m.

We also identified inactive gRNAs that had poor cleavage activity both *in vivo* and *in vitro*. In these cases, a gRNA might not effectively associate with Cas9 protein or it might associate with Cas9 but fail to form a cleavage-competent complex. We reasoned that in the latter case, inactive gRNAs could compete with active gRNAs for Cas9 protein binding. To test this idea, we compared cleavage with an active gRNA against its cognate site in the absence or presence of an equivalent amount of an inactive gRNA (Fig. 2a, inactive gRNAs 1–9). The majority of inactive gRNAs tested reduced the cleavage activity of the active gRNA compared with controls in which the same amount of random RNA or no competitor was added (Fig. 2b and Supplementary Fig. 4). The cleavage inhibition caused by inactive gRNAs was comparable to that of active gRNAs. Moreover, a 15-min incubation of the inactive gRNA with Cas9 protein before adding the active gRNA almost completely abolished cleavage activity (Fig. 2c). Competition between gRNAs also interferes with *in vivo* cleavage, both when Cas9 messenger RNA (Fig. 2d) or Cas9 protein are used (Supplementary Fig. 5). Interestingly, only one of the two inactive gRNAs that were tested *in vivo* competed efficiently, although both were comparable *in vitro* (Fig. 2b). These results indicate that many inactive gRNAs do not fail to complex with Cas9, rather they generate non-productive complexes both *in vitro* and *in vivo*, and can sequester available Cas9 protein away from productive gRNAs.

### Inactive gRNAs contain hairpins that inhibit cleavage

**Figure 3:**
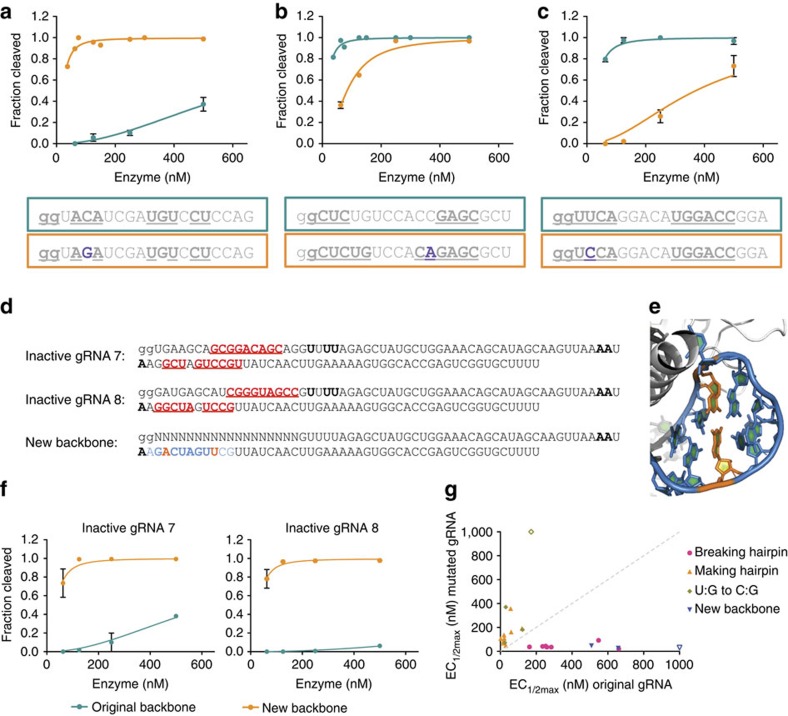
Internal hairpins in gRNAs reduce cleavage activity. (**a**) Breaking a gRNA interaction in a gRNA with low activity increases cleavage activity. The interacting region is indicated by bold and underlined text, whereas the single base-pair substitution is shown in purple. (**b**) Extending the potential hairpin of an active gRNA reduces cleavage activity. (**c**) Substitution of a G:U base pair with the canonical G:C base pair reduces cleavage activity. (**d**) Potential hairpins between the gRNA sequence and the constant region of the gRNA for two inactive gRNAs (Supplementary Table 2) and a mutated backbone that was designed to eliminate the interactions. (**e**) Substituted nucleotides in the new backbone shown in **d**, using pdb code 4OO8 (ref. 19) for visualization. (**f**) Cleavage activity of the two inactive gRNAs with the original and newly designed constant gRNA backbone. (**g**) Summary of the EC_1/2max_ differences for gRNAs with small numbers of substitutions that alter cleavage activity. The grey dashed *y*=*x* line represents the expected location of the points if the substitution did not have an effect on cleavage. Open shapes represent gRNAs with an EC_1/2max_ too high (>1,000 nM) to be accurately measured under tested conditions. At least two independent assays were completed for each *in vitro* cleavage experiment and error bars shown in all panels are s.e.m.

To understand the root cause of low activity, we compared inactive gRNAs with a large set of gRNAs with high activity. In cases where a similar but active gRNA could be identified, the differences between the two were dissected by testing chimeric gRNAs. This approach identified single base substitutions that rendered a previously inactive gRNA active (Supplementary Figs 6 and 7). For example, changing a C:G pairing between positions 13 and 20 to an A:G mismatch increased activity by over tenfold (Supplementary Fig. 6). Closer inspection of gRNA sequences revealed that the activating substitutions were located in potential gRNA hairpins in the target-specific portion of the gRNA. To further investigate whether internal gRNA interactions lead to a general reduction in activity, we introduced different mutations to modulate self-complementarity in 23 gRNAs and tested their activity. Substitutions that were predicted to eliminate or strengthen hairpins yielded the expected outcome: breaking predicted hairpins in low-activity gRNAs improved cleavage, whereas control substitutions in other areas of the gRNA did not (Fig. 3a and Supplementary Fig. 8). gRNAs with these activating substitutions were even able to cleave the originally targeted site *in vivo*, if the genomic mismatch was not within the gRNA seed sequence^17^ (Supplementary Fig. 9).

Not every gRNA containing a predicted hairpin sequence showed low *in vitro* cleavage activity. We hypothesized that these hairpins might not be sufficiently energetically favourable to form within the Cas9–gRNA complex. Indeed, adding an additional base pair of complementarity reduced the activity of these gRNAs (Fig. 3b and Supplementary Fig. 10). Analogously, substantially reduced activity also resulted when gRNA sequences containing hairpins with G:U wobble base pairings (determined with Mfold^18^) were modified to replace the G:U with a stronger G:C base pair (Fig. 3c and Supplementary Figs 11 and 12). These results are consistent with the idea that strong internal gRNA interactions can interfere with Cas9 activity.

Although most inactive gRNAs studied contained predicted hairpins within the gene-specific portion of the gRNA, several were instead predicted to have interactions between the gene-specific sequence and the gRNA backbone (Fig. 3d and Supplementary Fig. 13). To test whether these interactions reduce activity, we used a Cas9–gRNA crystal structure^19^ to guide the introduction of complementary substitutions in the backbone area that were predicted to eliminate the interaction but not interfere with the gRNA binding to Cas9. Strikingly, these mutations dramatically improved cleavage activity (Fig. 3e,f and Supplementary Figs 13 and 14). For example, the replacement of a C and G in the backbone disrupted detrimental interactions with a G at position 13 and a C at position 17, respectively, resulting in a 19-fold increase in activity. Taken together, the consistent decrease or increase of Cas9 activity on making or breaking gRNA hairpins, respectively, highlights the importance of minimizing these interactions as a general principle of gRNA activity (Fig. 3g). To facilitate incorporation of this principle into pipelines for gRNA production, we included a supplementary Python script (Supplementary Software) that screens gRNAs for possible internal interactions and have incorporated these rules into CHOPCHOP^20^, a popular publically available web server for gRNA design. We assessed the improvements to prediction of gRNA success using our own data and in relationship to previously published data sets and algorithms^6^,^10^,^11^ (Supplementary Fig. 15) and found that hairpins are enriched in low-activity gRNAs across multiple data sets and are sometimes missed by published algorithms.

**Figure 4:**
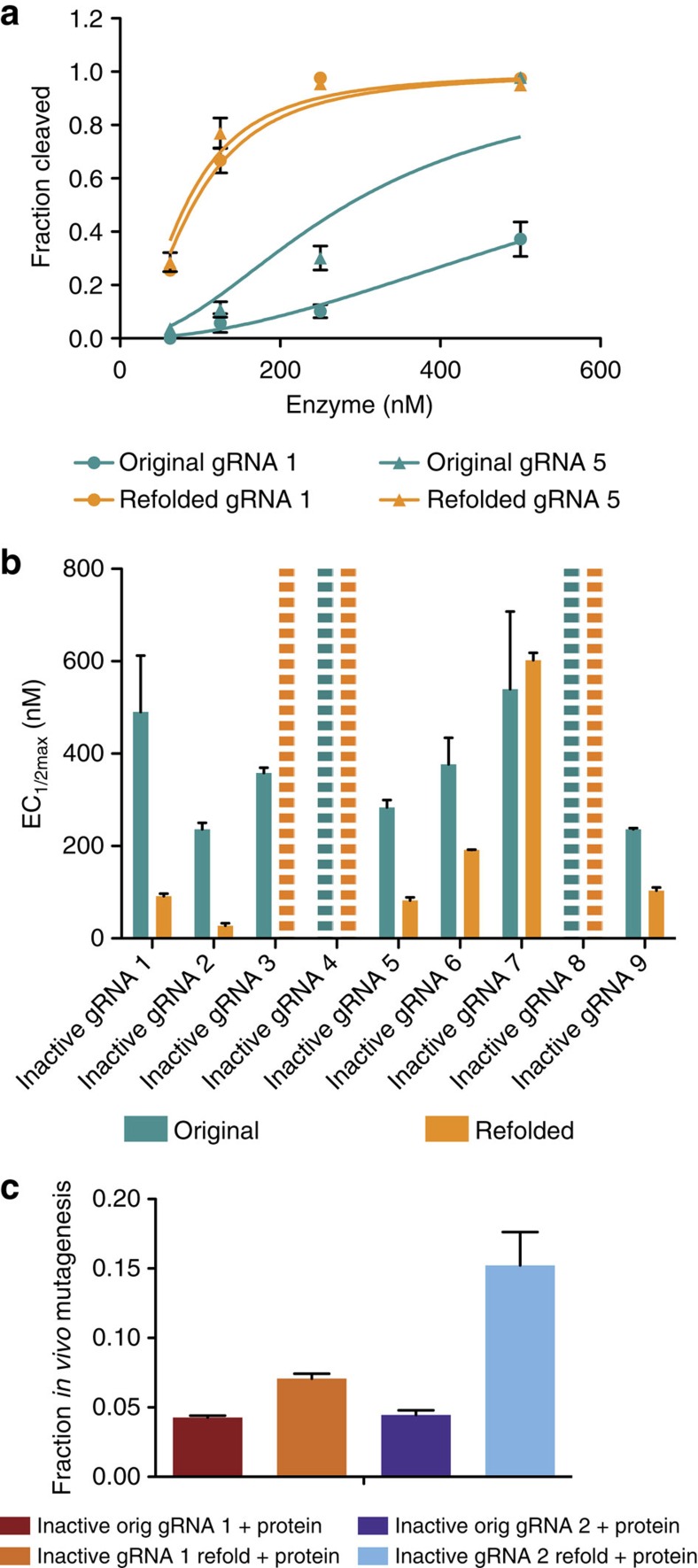
Refolding of inactive gRNAs can increase cleavage activity. (**a**) Cleavage traces for two gRNAs before and after refolding. (**b**) EC_1/2max_ values for nine inactive gRNAs (Supplementary Table 2) before and after refolding. Dashed bars represent gRNAs with an EC_1/2max_ too high (>1,000 nM) to be accurately measured under tested conditions. (**c**) Cleavage activity *in vivo* for two inactive gRNAs, before and after gRNA refolding, combined with 13 μM Cas9 protein (0.5 nl of 6.5 μM injected). Although both cleave more efficiently *in vivo* after gRNA refolding, inactive gRNA 2 is more responsive to the procedure. At least two independent assays were completed for each mutagenesis or *in vitro* cleavage experiment and error bars shown in all panels are s.e.m.

RNAs can form internal structures such as hairpins when the RNA folds concurrently with its transcription^21^. We therefore reasoned that transcription might lead to inactive gRNA structures and hypothesized that refolding of the gRNA could correct these detrimental interactions if the active form is thermodynamically favourable. Indeed, heating and slowly cooling some gRNAs before cleavage assays resulted in substantial activity increases (Fig. 4a). For example, refolding of inactive gRNA 1 resulted in a sevenfold increase in activity. Over half of the inactive gRNAs were responsive to this procedure (Fig. 4b and Supplementary Fig. 16) and activity was improved *in vivo* as well (Fig. 4c and Supplementary Fig. 17). Refolding substantially worsened the activity of one gRNA, inactive gRNA 3, which indicates that the inactive structure is more energetically favourable than the cleavage-competent structure. These results provide additional evidence that gRNA secondary structure can interfere with its activity and suggest that some inactive gRNAs can be rescued by refolding them after transcription.

## Discussion

In summary, our study of the *in vivo* and *in vitro* activities of gRNAs provides two major conclusions as follows:

First, short genomic elements can be refractory *in vivo* to gRNAs that are active *in vitro*. The factors determining whether an active Cas9 complex can cleave *in vivo* are still unclear but one possibility is that high-affinity interactions of protein complexes with gRNA target sites prevent access of the Cas9–gRNA complex. Despite this potential roadblock to gRNA activity, we found that at the genomic sites we tested, refractory regions were short, allowing the use of neighbouring gRNAs to target a specific locus. Our results identify candidate factors such as the CTCF genome-organization factor as potential recognition or cleavage inhibitors, opening the door to future improved prediction and avoidance of refractory regions. More detailed analyses of regions that cannot be readily cleaved could also lead to insights into the three-dimensional structure of chromatin or to the discovery of additional molecules that block access to the genome.

Second, internal gRNA interactions hinder the formation of a cleavage-competent complex and compete with active gRNAs for binding to Cas9 protein, both *in vitro* and in a developing zebrafish embryo (Fig. 2). Our *in vivo* competition data indicate that considering the ratios of gRNAs in multiplexing experiments, in particular when as many as ten gRNAs are tested in the presence of limited levels of Cas9 protein^11^, is critical to optimize the cleavage activity of all members of the pool. However, not every gRNA that competitively binds to Cas9 *in vitro* elicits comparably reduced *in vivo* mutagenesis. This observation suggests that additional features of the *in vivo* system influence cleavage, including the stability of the Cas9–gRNA complex^11^, the stability of the gRNA itself and the potential exchange between active and inactive gRNAs (Supplementary Fig. 5) during the longer timescale of *in vivo* experiments.

Detrimental internal interactions in gRNAs may be restricted to the target-specific portion of the gRNA or may form between the target-specific portion and distant regions of the gRNA backbone. The gRNA backbone structure contains four regions of internal interactions that stabilize its interaction with the protein. Notably, the backbone region (Fig. 3e and Supplementary Fig. 14) prone to interactions with the target-specific portion of the gRNA also contains fewer base pairs than the other regions. This observation suggests that the inherent instability of this region makes it more susceptible to interactions with the target-specific portion. We suggest that both types of misfolded gRNAs can bind to Cas9 through the remaining correctly folded backbone structures, but that the target-specific portion is prevented from proper interaction with DNA target sites. Strikingly, refolding such gRNAs or making single base-pair substitutions is often sufficient to improve Cas9 cleavage activity: gRNAs with mismatches can cleave *in vivo* (Supplementary Fig. 9) and refolded gRNAs can become active.

Although we have provided direct evidence that intra-gRNA interactions can inhibit cleavage, it is still challenging to accurately predict which hairpins will be detrimental (Supplementary Figs 10,15 and 18). Although we find that Mfold prediction of the free energies of these interactions sometimes accurately mirrored the observed experimental trends, there are limitations to this approach (Supplementary Fig. 18) that might be due to changes to the RNA folding energetics within the Cas9-binding site. Our findings point to the need for improved algorithms for prediction of RNA structures, in particular in the context of RNA–protein complexes^22^,^23^. Clarifying our understanding of the biophysical basis for Cas9–gRNA selectivity and how a gRNA incorporates into the Cas9 protein is an important step in optimizing the system and accurately predicting gRNA activities. In conclusion, our study not only increases our understanding of Cas9–gRNA complex formation, but also identifies additional rules for gRNA design and provides new approaches to confer activity to seemingly inactive gRNAs.

## Methods

### Production of gRNAs

Templates for gRNA transcription with T7 polymerase were generated by two-oligo PCR, using one oligo containing the T7 promoter and gene-specific sequence with overlap to a second reverse complement oligo containing the constant region of the gRNA backbone. An optimized gRNA constant region was used^24^, except in Supplementary Fig. 13 where two constant regions are compared for one gRNA. As in previous work^8^,^25^, the bases at the first two positions in the gRNA were always substituted for guanine to accommodate preferences of T7 RNA polymerase. The product was purified with the E.Z.D.A. Cycle-Pure kit (Omega Bio-tek, Norcross, GA). All gRNAs were transcribed with the Megascript T7 kit (Thermo Fisher Scientific, Waltham, MA), using half-size (10 μl) reactions and DNase treated. Purification of the gRNA was either done with ammonium acetate/ethanol precipitation or with column purification, using RNA Clean & Concentrator columns (Zymo Research, Irvine, CA), if precise quantification of gRNA concentration was required (Fig. 2). For column-purified gRNAs, RNA concentration was determined using a Nanodrop spectrophotometer and all gRNAs were visualized with agarose gel electrophoresis. The random RNA control was concentration matched to gRNAs and similarly sized, as well as equivalently column purified.

### Fish husbandry and microinjection

All protocols and procedures involving zebrafish were approved by the Harvard University/Faculty of Arts & Sciences Standing Committee on the Use of Animals in Research and Teaching (IACUC; Protocol #25-08).

Zebrafish TLAB embryos were collected and injected at the one-cell stage with 300 pg of mRNA of Cas9 mRNA and excess gRNA^5^,^8^ (∼300–500 pg). For *in vivo* competition experiments with Cas9 mRNA, 50 pg of active gRNA was used, to more accurately replicate experimental conditions in which five or more gRNAs are multiplexed.

### Zebrafish somatic mutagenesis data

Zebrafish genomic DNA was extracted from pools of 10–20 embryos at 24–30 hpf, using the HotSHOT method^26^. Mutagenesis rate was determined using MiSeq sequencing, as previously described^8^, by dividing the number of reads with mutations at the gRNA cleavage site by the sum of these reads and those that maintain the wild-type sequence. The large set of gRNAs determined to be active and used as a comparison for low activity guides were previously published^8^ or assessed using T7 endonuclease assays, MiSeq sequencing or germline transmission data. At least two independent assays were completed for each mutagenesis experiment and error bars shown in all figures are s.e.m. All experiments in which gRNAs are being compared with each other were conducted with the same preparation of Cas9 mRNA, to minimize differences due to overall mRNA activity and quality, and the injections were conducted side-by-side on the same day.

### Cas9 cleavage assays

His-tagged Cas9 endonuclease (Addgene plasmid 47327) was produced as follows^8^, using auto-induction^27^, growing for 12 h at 37 °C, followed by 24 h expression at 18 °C. Cells were lysed with sonication and Cas9 protein was purified with Nickel-NTA resin (G-Biosciences), using 20 mM Tris pH 8, 30 mM Imidazole and 500 mM NaCl for washes, and 20 mM Tris pH 8, 500 mM Imidazole and 500 mM NaCl for elution. Protein was buffer exchanged into 20 mM Tris, 200 mM KCl and 10 mM MgCl_2_, concentrated and aliquots were stored at −80 °C. The protein was confirmed to be of >95% purity by SDS–PAGE and Coomassie staining. All cleavage assays were done under single-turnover conditions^1^,^13^. Twofold serial dilutions of enzyme (between 600 and 31 nM, depending on the experiment and gRNAs being tested) were made in a reaction buffer consisting of 200 mM KCl, 10 mM MgCl_2_ and 20 mM Tris-HCl pH 8.0. These enzyme dilutions were combined 3:1 with ∼10 nM of substrate, typically 3 μl of Cas9 endonuclease and 1 μl of 50 ng μl^−1^ substrate. This mix of protein and target was immediately combined 4:1 with excess gRNA, unless otherwise noted. The substrates used were column-purified PCR products (1,400–2,000 nucleotides), amplified from target site arrays that were cloned into a plasmid backbone.

Reactions were run for 30 min at 37 °C and halted with a reaction stop buffer, followed by 80 °C incubation for 5–10 min. Adding the stop buffer results in a final concentration of ∼15 mM EDTA, 7.5% glycerol, 0.5% SDS and bromophenol blue. The resulting cleavage products were separated by gel electrophoresis on 1.8–2% agarose tris/borate/EDTA (TBE) gel and were visualized by staining with ethidium bromide. Fraction cleavage was calculated by dividing the density of product bands by the total of the product and substrate band densities, as previously described^13^,^28^,^29^. The spectral densities were quantified using ImageJ and the fraction cleavage was plotted versus Cas9 endonuclease concentration using GraphPad Prism. The EC_1/2max_ was calculated by fitting the fraction cleavage to a sigmoid function, as previously described^28^, using a value of 2 for the Hill coefficient. At least two independent assays were completed for each cleavage experiment and error bars shown in all figures are s.e.m.

### gRNA refolding

gRNAs, resuspended in water after ethanol precipitation, were heated to 98 °C for 2 min and temperature was lowered at a rate of 0.1 °C s^−1^ until 30 °C was reached.

### gRNA interaction algorithm

To facilitate avoidance of internal gRNA interactions, we have included a Python script (Supplementary Software) and incorporated the same rules into the CHOPCHOP^20^ web server. The development server for CHOPCHOP is available at the following web address: https://chopchop.rc.fas.harvard.edu/dev/.

To use the attached script, the name of a file containing the 20 nucleotides of gRNA sequence, with each gRNA on a new line, is passed to the script with the -f option. If the first two bases of the gRNA are going to be experimentally substituted for guanine, as in this work, the -gg option is used. The -bb option needs to be included if an extended gRNA backbone^24^ is used, as in this work. The script output provides a list of the gRNAs with hairpin sequences.

### Identification of sequence-similar genomic sites

Sites containing sequences complementary to *in vivo* inactive gRNAs (Fig. 1e and Supplementary Fig. 2) were identified using a previously published^29^ Position Specific Scoring Matrix search algorithm (https://github.com/justinashworth/pssm). This programme minimizes the number of substitutions in a given sequence. For identifying CTCF-containing sites, searches were completed both to find matches for the full *in vivo* inactive gRNA 3 target and for just the putative CTCF region (Supplementary Table 1). An example of Position Specific Scoring Matrix, the one used for finding putative CTCF-containing sites, is available in the Supplementary Methods. Several target sites were chosen blindly from among the top-matched candidates that were identified in the Zv9 version of the zebrafish genome.

### Data availability

The data that support the findings of this study are available from the corresponding author on reasonable request. The sequencing data that support the findings of this study are available from NCBI Sequence Read Archive with the accession code SRP073406.

## Additional Information

**Accession codes:** All relevant MiSeq sequencing data has been deposited in NCBI Sequence Read Archive and can be accessed using SRA accession number SRP073406.

**How to cite this article:** Thyme, S. B. *et al*. Internal guide RNA interactions interfere with Cas9-mediated cleavage. *Nat. Commun.* 7:11750 doi: 10.1038/ncomms11750 (2016).

## Supplementary Material

Supplementary Information Supplementary Figures 1-18, Supplementary Tables 1-4 and Supplementary Methods

Supplementary Software Python script to calculate whether a gRNA could contain internal interactions, both within the gene-specific region and between the gene-specific region and gRNA backbone. Use of the script is described in detail in the Methods.
