# Supplementary material for: Internal guide RNA interactions interfere with Cas9-mediated cleavage
Source: Nat Commun. 2016 Jun 10;7:11750. doi: 10.1038/ncomms11750 (PMC4906408; doi:10.1038/ncomms11750)
Supplement: Supplementary Information — Supplementary Figures 1-18, Supplementary Tables 1-4 and Supplementary Methods [file ncomms11750-s1.pdf]

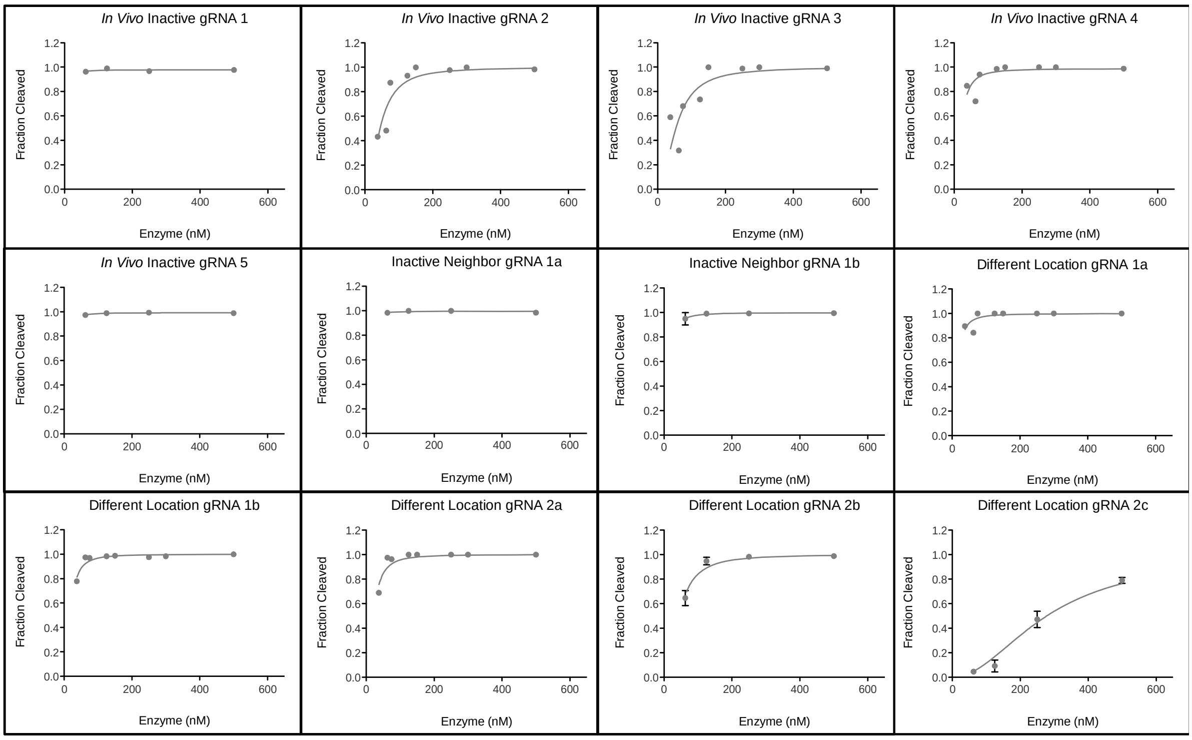

Supplementary Figure 1. Full cleavage data for gRNAs with high *in vitro* activities that induce low rates of mutagenesis (*in vivo* inactive gRNAs). Target site cleavage traces for gRNAs described in Figure 1.The gRNAs labeled "Inactive" are the original five that were identified to have high *in vitro* activity but low *in vivo* activity (Fig. 1a). Those labeled "Neighbor" are the ones that are neighbor the *in vivo* inactive gRNA 1 and induce low levels of *in vivo* mutagenesis (Fig. 1b, a is on left of original gRNA, b is on right). Those labeled "Different Location" are the gRNAs that contain sequences from two of the original five (1 and 2), but are at different locations in the zebrafish genome (Supplementary Fig. 2).

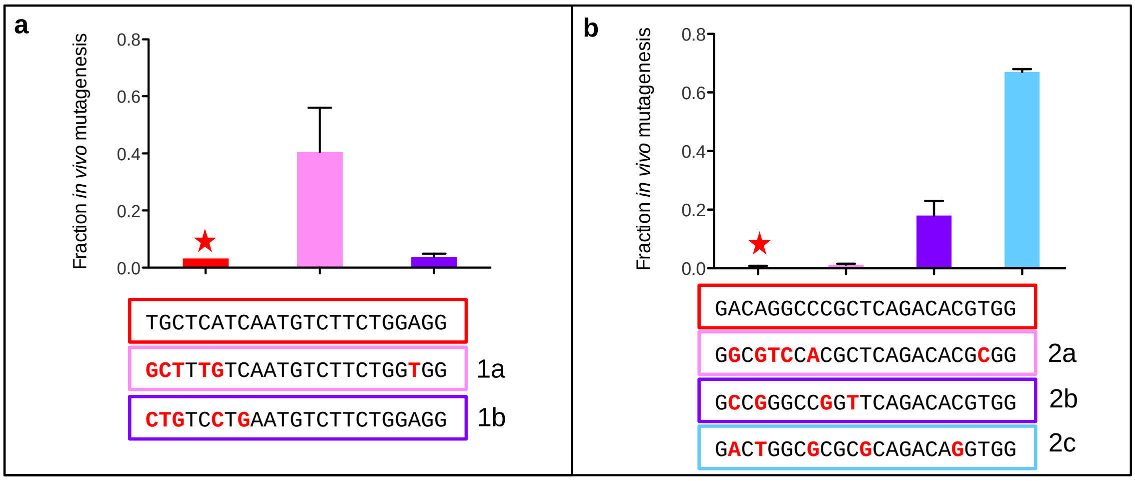

Supplementary Figure 2. *In vivo* cleavability of sites containing sequences matching *in vivo* inactive gRNAs 1 and 2. Sites are labeled to correspond to *in vitro* cleavage data in Supplementary Fig. 1. Targets sites inducing low mutagenesis have high cleavage *in vitro* (Supplementary Fig. 1). It is currently not known what genomic factors are binding to these sites/sequences. The DNA target at the different genomic location is shown with substitutions from the original sequence in red. (**a**) Mutagenesis of zebrafish embryos for sites containing sequences matching *in vivo* inactive gRNA 1. (**b**) Mutagenesis of zebrafish embryos for sites containing sequences matching *in vivo* inactive gRNA 2.

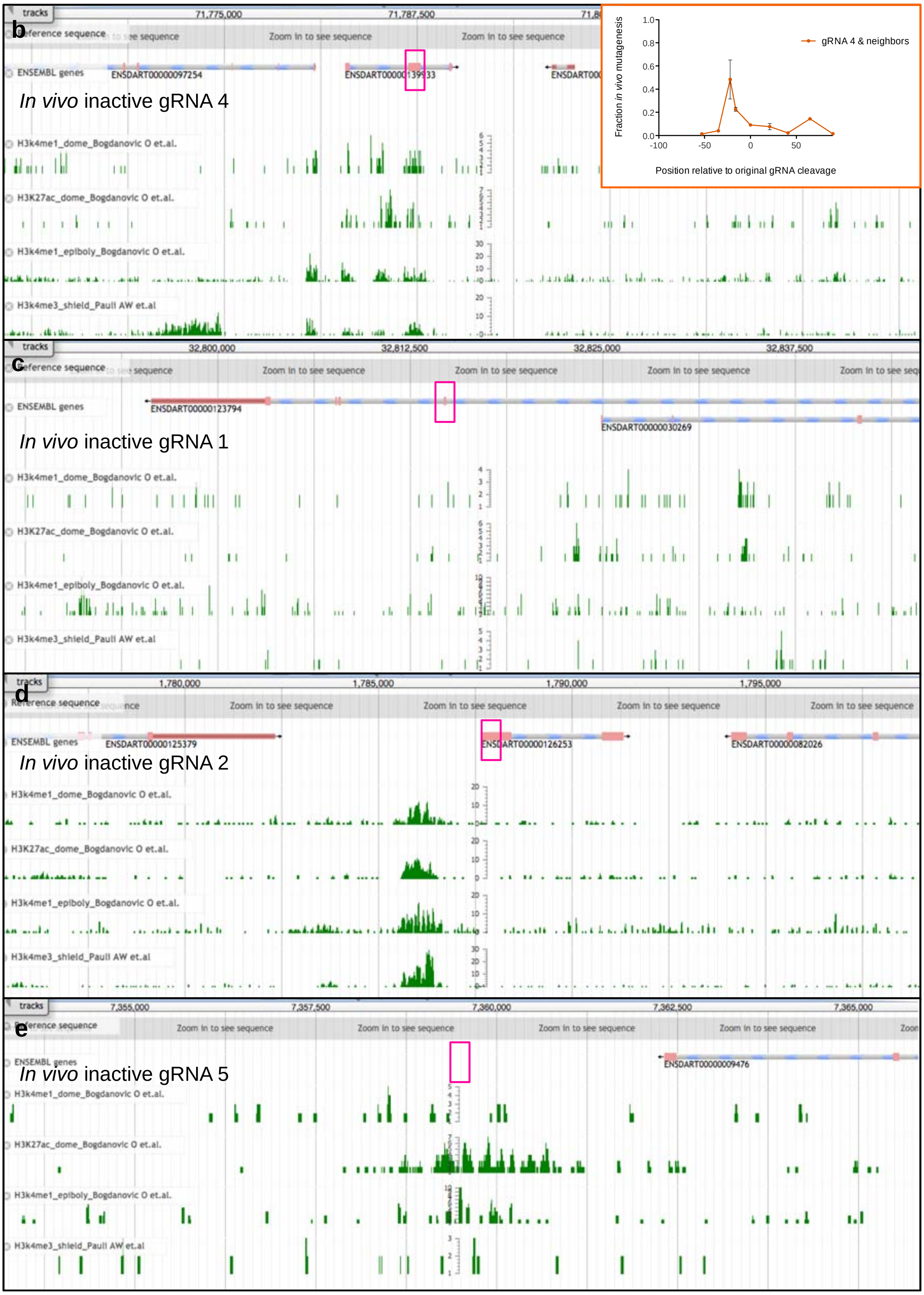

Supplementary Figure 3. Histone marks in early zebrafish development near *in vivo* inactive gRNA sites. (**a**) Plot of *in vivo* cleavage for gRNAs neighboring inactive gRNA 4, with the distance relative to original cleavage site. (**b-e**) Plots of published chromatin marks, made using the ZENCODE genome browser, near *in vivo* inactive gRNAs, with the gRNA region marked in pink, in the area of (**b**) *in vivo* inactive gRNA 4, (**c**) *in vivo* inactive gRNA 1, (**d**) *in vivo* inactive gRNA 2, and (**e**) *in vivo* inactive gRNA 5. *In vivo* inactive gRNA 3 is not shown but is similar to gRNAs 1 and 2, lacking notable peaks for these histone marks near the cleavage site.

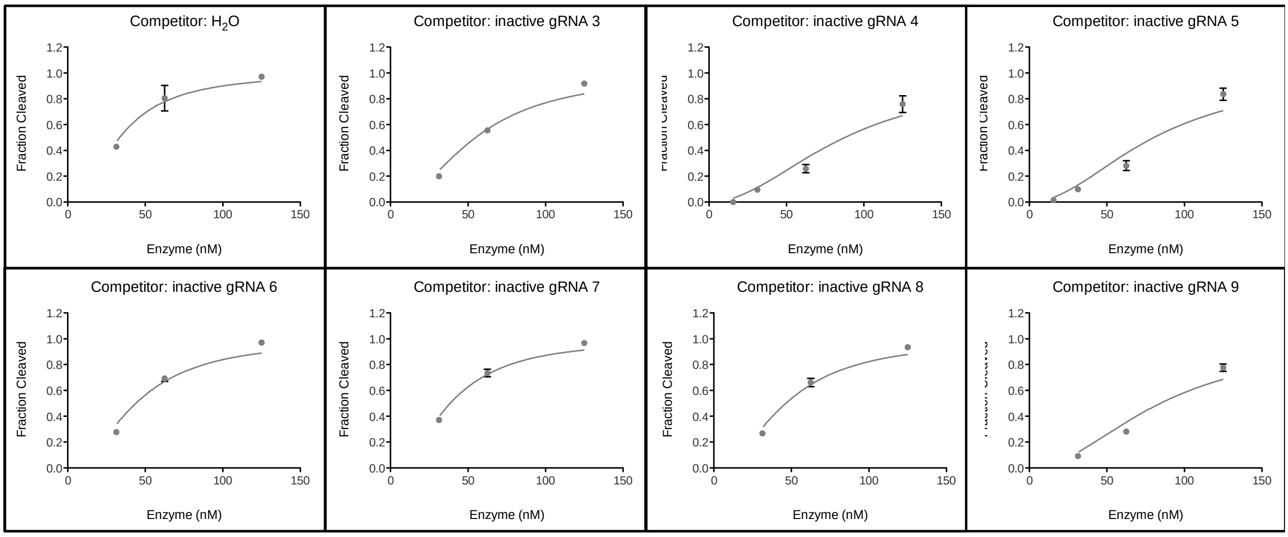

Supplementary Figure 4. Full cleavage data for enzyme-limited gRNA competition experiment. Target site cleavage traces for gRNAs shown in Fig. 2b (except those already shown in Fig. 2a). Equal amounts of competitor, either inactive gRNA or control sample, are combined with an active gRNA and the cleavage shown is for the active gRNA with its cognate site.

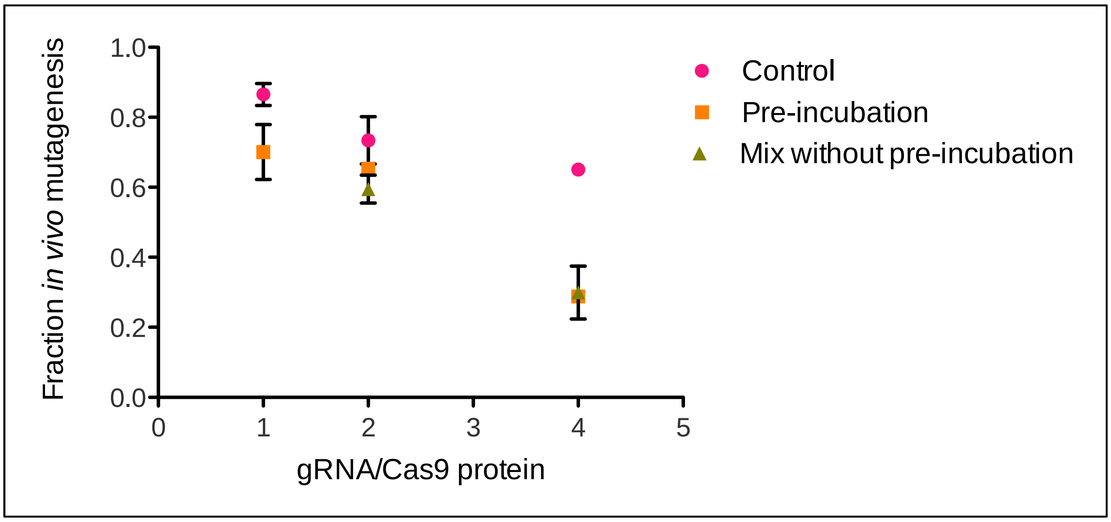

Supplementary Figure 5. *In vivo* competition experiment with varying amounts of Cas9 protein. This experiment demonstrates that the effect of competition *in vivo* depends on how much Cas9 protein is available. Here we combined (1:1) 600 pg (~13 µM) of active gRNA 3 (Supplementary Table 3) with 13 µM of Cas9 enzyme and injected 1 nL (300 pg gRNA, 6.5 µM Cas9) for the condition where gRNA/Cas9 is equal. We found little effect of competition at these ratios, even when the inactive gRNA was pre-incubated at room temperature for five minutes with the Cas9 protein. This indicates that there is could some exchange between active and inactive gRNAs over the time they are in the embryo because the Cas9 protein should be limiting under these conditions. However, when the Cas9 protein is reduced by four-fold and the gRNA amount is kept constant, there is a substantial effect of competition with the inactive gRNA compared to the control injections. Pre-incubation and mixing of the gRNAs at the same time do not result in substantially different outcomes for any condition, supporting the idea that there could be some gRNA exchange occurring in the embryo.

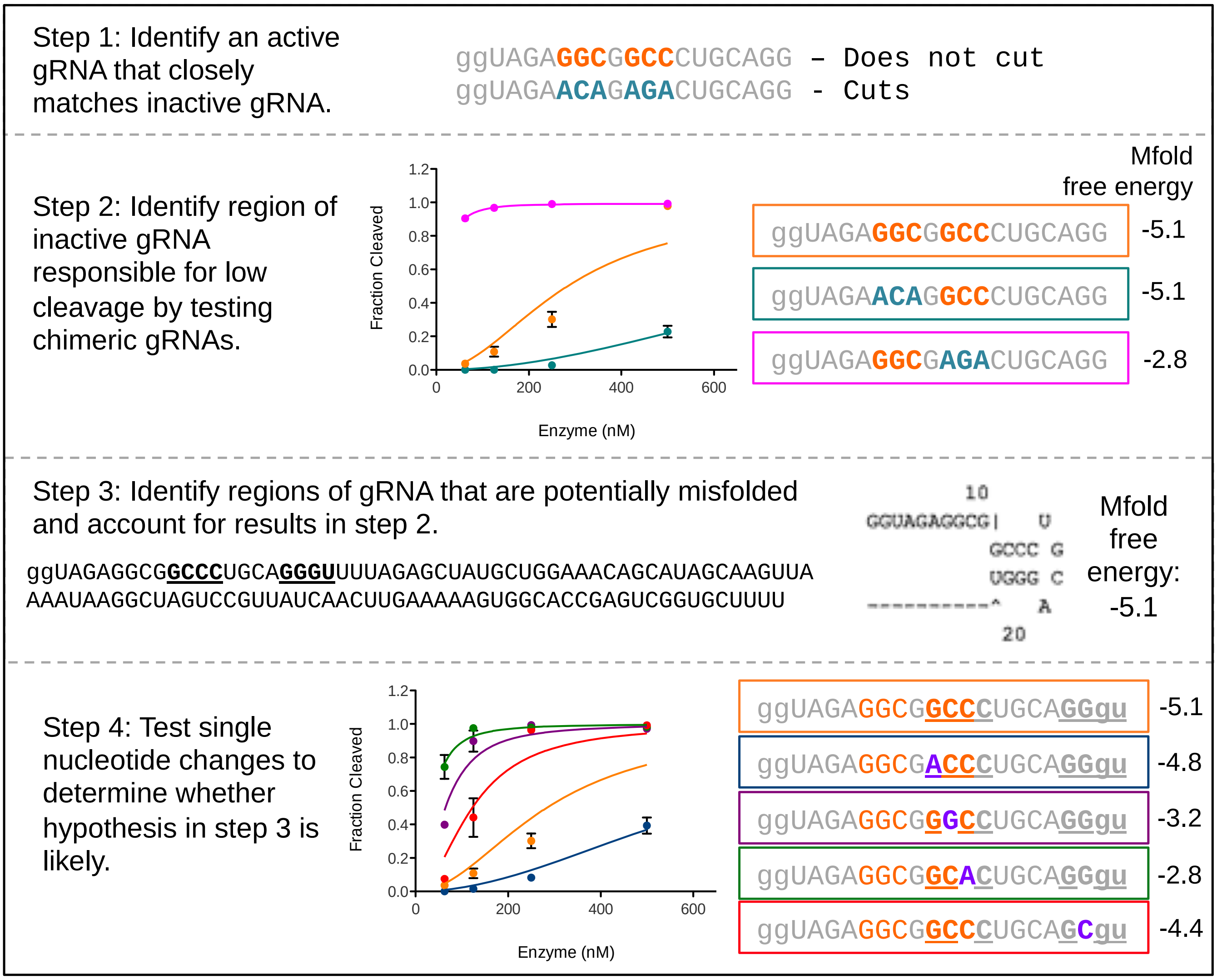

Supplementary Figure 6. Comparing inactive and active gRNAs to identify features influencing catalysis. First, an active gRNA is found that is similar to the inactive gRNA being studied, by comparing the sequence to a large set of active gRNAs tested in our lab. Then, chimeric gRNAs are tested against cognate chimeric target sites to identify which regions of the inactive gRNA are responsible for low activity. In this case, inactive gRNA 5, a putative hairpin was identified mainly in the gene-specific portion of the gRNA, but also with the first two bases of the gRNA backbone. Single nucleotide substitutions (purple) within the putative hairpin (underlined) showed varying levels of increased activity over the original gRNA, likely depending on their importance to the formation of the hairpin. Mutations on both sides of the hairpin had the same effect, indicating that it is forming between the predicted regions. Mfold free energies were collected for this set of gRNAs, as well as for all subsequent calculations except in those in Figure S13, for the entire gene-specific portion and the first “GU” of the gRNA backbone because we found for this gRNA that it can interact with the gene-specific portion. The predictions are not done with the entire backbone because the prediction programs are not aware of the Cas9 enzyme and that it is energetically favorable to form the correct backbone configuration to bind to the protein. We found that many unlikely structures were predicted when the entire backbone included in the calculation.

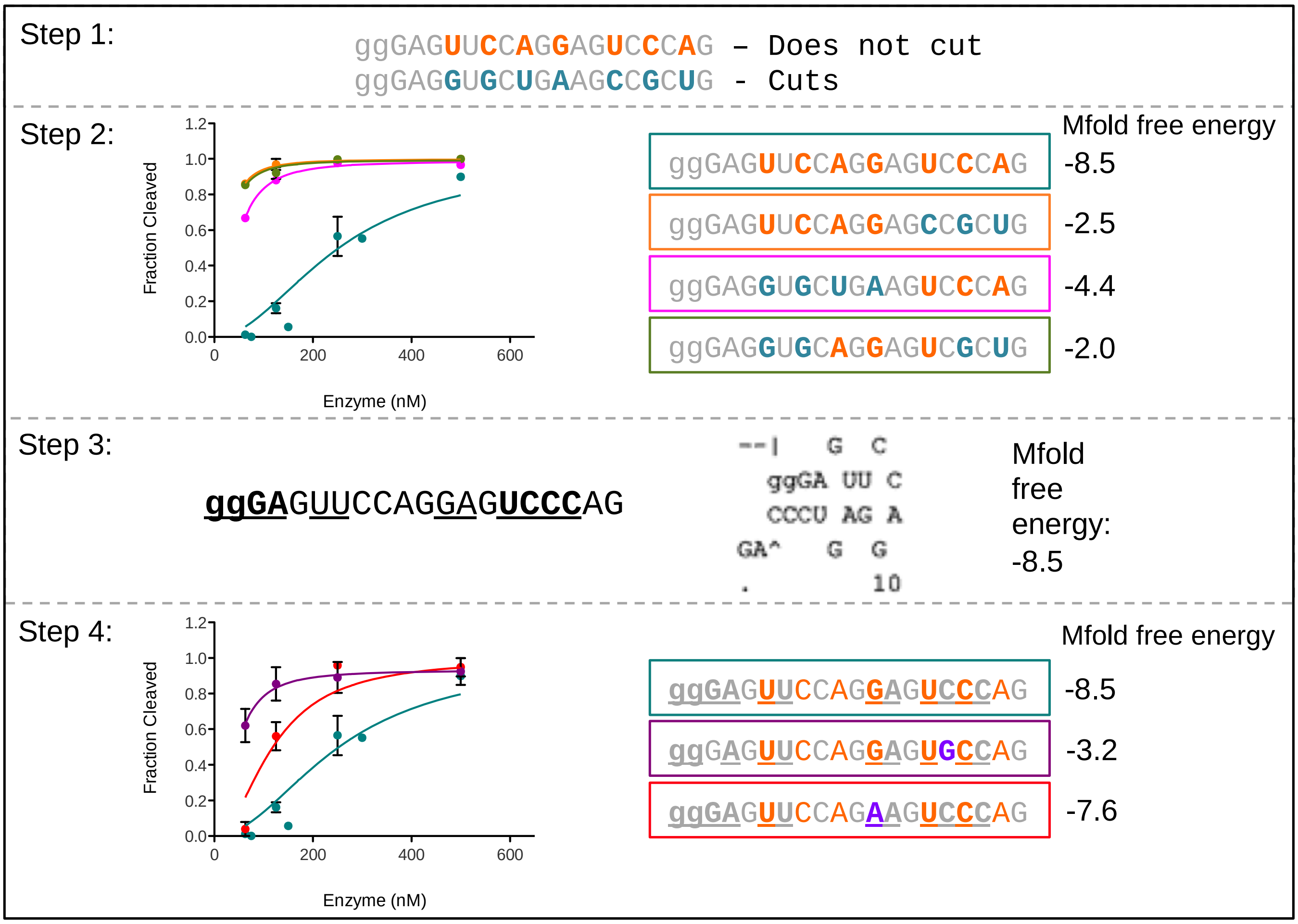

Supplementary Figure 7. Second example of comparing inactive and active gRNAs. The steps followed in Supplementary Fig. 6 were followed for other gRNAs. For this particular inactive gRNA, there are two putative regions of internal gRNA interactions, both underlined in steps 3 and 4. Mutations in either of the two regions result in increased activity, although the substitution in the longer interacting region resulted in a higher increase than the other.

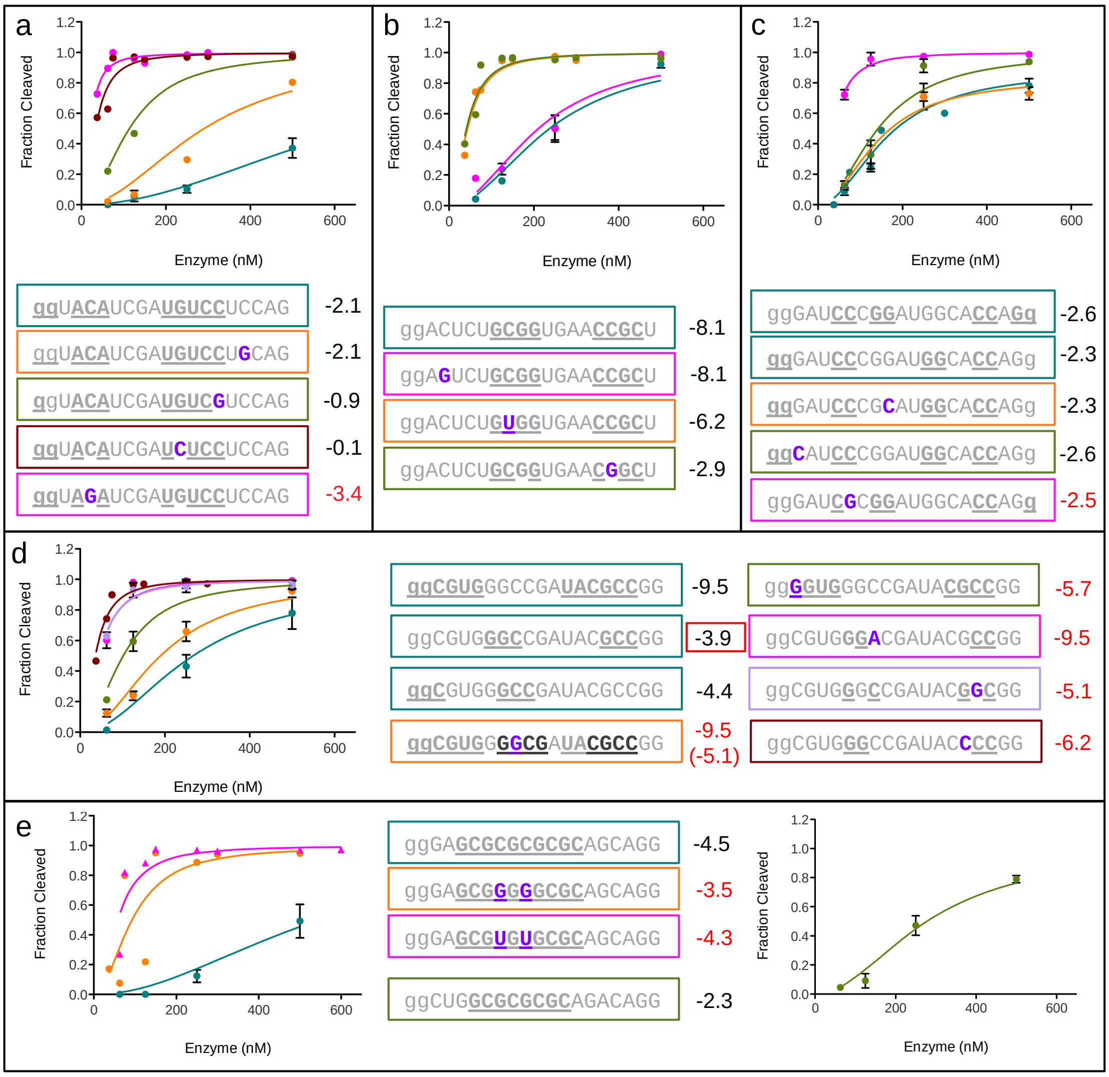

Supplementary Figure 8. Full cleavage data for gRNAs where hairpins were broken with substitutions. Target site cleavage traces for gRNAs shown in Fig. 3. The wild-type sequence is displayed on top, boxed with teal (multiple boxes if multiple possible hairpins). Putative hairpins were predicted (underlined and bold) and substitutions (purple) were made. For all cases, the Mfold free energy was predicted, and the energies are shown in red when the predicted lowest free energy does not follow the expected trend. These discrepancies could be due to altered energetics of folding within the Cas9 active sites, as the lowest free energy predicted hairpin may not be favorable in this context, and the structures described in each case are examined in detail in Supplementary Fig. 15e-h (corresponding to a, d, c, and e in this figure). **a**) A completely different hairpin structure was predicted for the last, best-cleaving, gRNA (pink) with a lower free energy, and it is unlikely that this hairpin is plausible. **b**) Mfold gives G:U pairings better free energies than they appear to have experimentally (-6.2 vs. -2.9, both cleave the same), but otherwise the prediction is reasonable. **c**) Two hairpins are possible, although the substitution that breaks the one with worse free energy (-2.3) shows the best experimental cleavage and Mfold identifies a completely different hairpin with a comparably low free energy for this best-cleaving gRNA (likely not forming). **d**) The finding that the gRNA boxed in pink is high-cleaving conflicts with the lowest free energy predicted hairpin (-9.5). There are two plausible explanations: 1) the hairpin with free energy -9.5 is not forming and instead the less stable hairpin with free energy -3.9 is forming. This -3.9 hairpin is compatible with all data, if a new and different hairpin forms for the gRNA boxed in orange (new hairpin in darker grey, free energy -5.1). 2) The C to A substitution in the gRNA boxed in pink is not compatible with forming the -9.5 hairpin for some unknown reason, such as space within the Cas9 binding site. **e**) A repetitive stretch of GCs (underlined and bold) could be forming internal interactions, as activity increased when the stretch was broken. However, a second gRNA with a similar stretch of GCs was also low activity although the region was potentially too short to form internal gRNA interactions. It is possible that the repetitive nature of the sequence in this case was responsible for low activity, rather than hairpins.

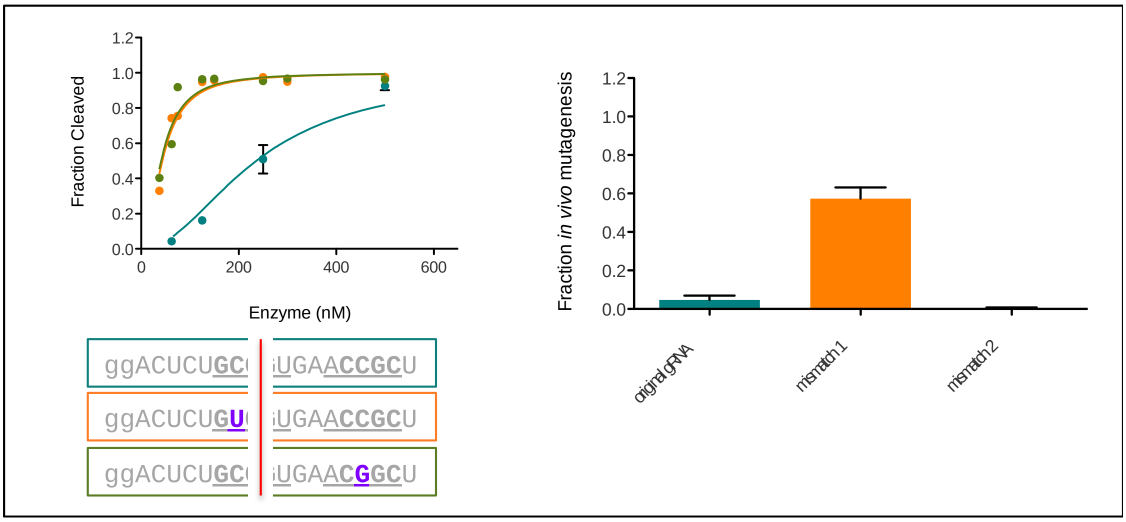

Supplementary Figure 9. gRNAs with single substitutions can cleave mismatch target sites *in vivo*. Single nucleotide substitutions in a predicted hairpin were found to increase the cleavage activity of inactive gRNA 2. When these two gRNAs were tested in zebrafish embryos against the original target site, one of the two showed higher *in vivo* cleavage activity than the original gRNA. The nucleotide substitution in the gRNA with high activity was far from the PAM and not within the seed sequence of the gRNA. The seed sequences (10 nucleotides closest to PAM) are separated from the other regions of the gRNAs with a red line.

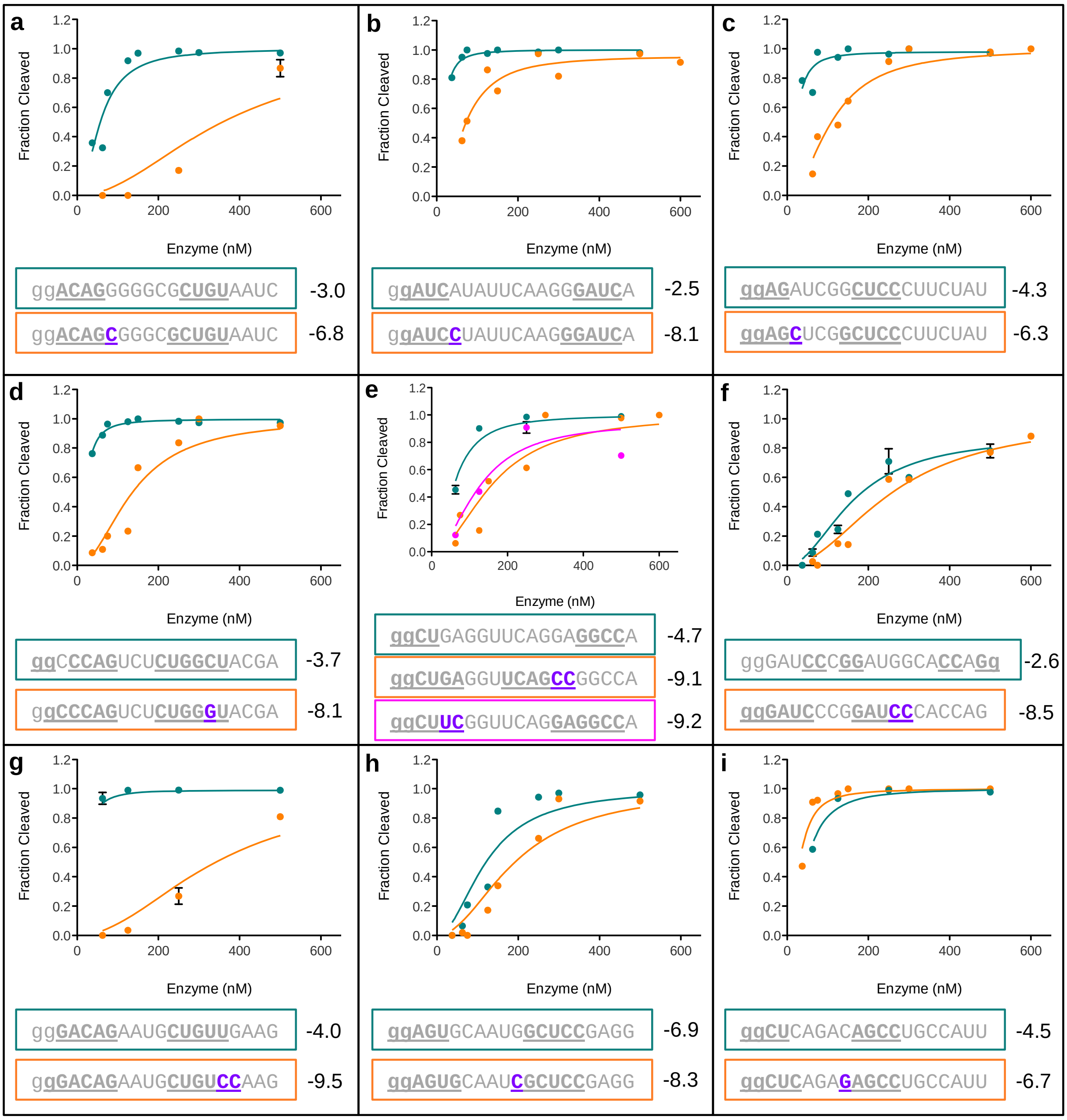

Supplementary Figure 10. Full cleavage data for gRNAs where hairpins were made with substitutions. Target site cleavage traces for gRNAs shown in Fig. 3. gRNAs were tested with either one or two nucleotide substitutions that were predicted to increase the strength of a hairpin. Three of the gRNAs did not have strongly reduced activity from the added interactions (**f**, **h**, **i**), however two of these gRNAs already had relatively poor cleavage activity (**f**, **h**) without the substitutions and were likely already forming detrimental internal gRNA interactions.

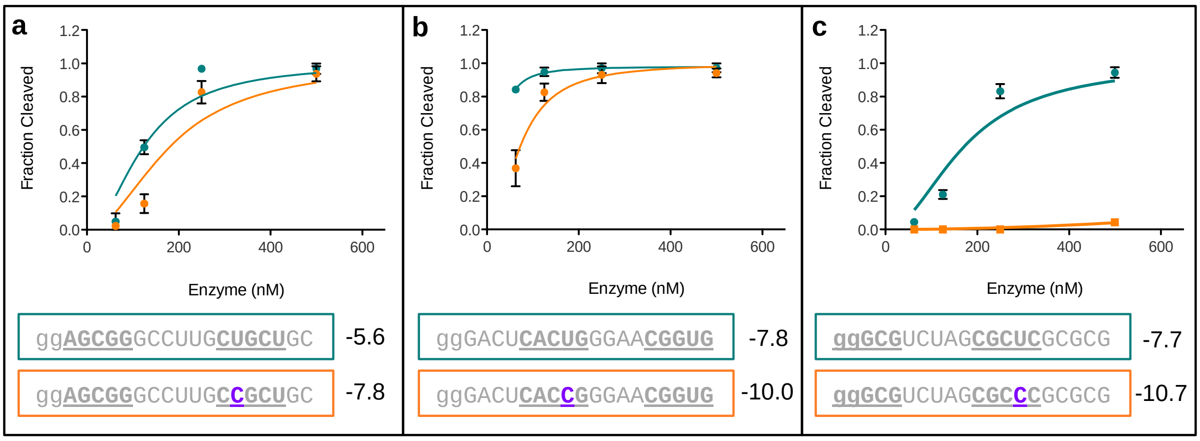

Supplementary Figure 11. Full cleavage data for gRNAs where hairpins were made by changing G:U to G:C pairing. Target site cleavage traces for gRNAs shown in Fig. 3. These gRNAs were predicted to contain strong hairpins with Mfold, but they showed relatively high *in vitro* cleavage activity. Changing the G:U pairing to a G:C resulted in reduced cleavage activity.

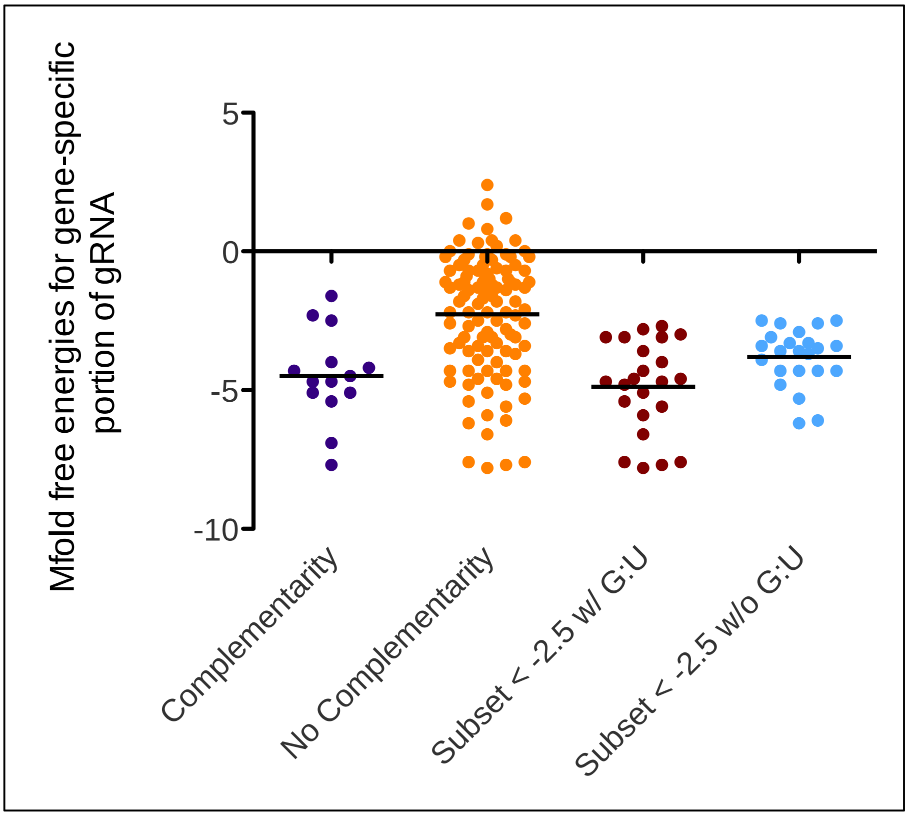

Supplementary Figure 12. Comparison of Mfold free energies and intra-gRNA complementarity. The biggest distinction between prediction with Mfold and a simple calculation of complementarity, defined as four or more bases that can interact within the gene-specific region, is the low free energy Mfold prediction for G:U base-pairing. Breaking down the gRNAs that do not contain obvious complementarity, but are predicted to have low free energies with Mfold (examined all with free energies less than -2.5), we find that those with the lowest free energies all contain predicted G:U pairings (unpaired t test p = 0.0114, comparing the two subsets with free energies <-2.5). As we show experimentally (Fig. 3c, Supplementary Fig. 11), the substitution of a G:U pairing for a G:C pairing can significantly strengthen a hairpin and reduce activity. The remaining gRNAs with low Mfold-predicted free energies and without G:U pairings often contained a hairpin with just three base-pairs or a hairpin with a bulge in the sequence that may not be compatible with folding in the Cas9 binding site.

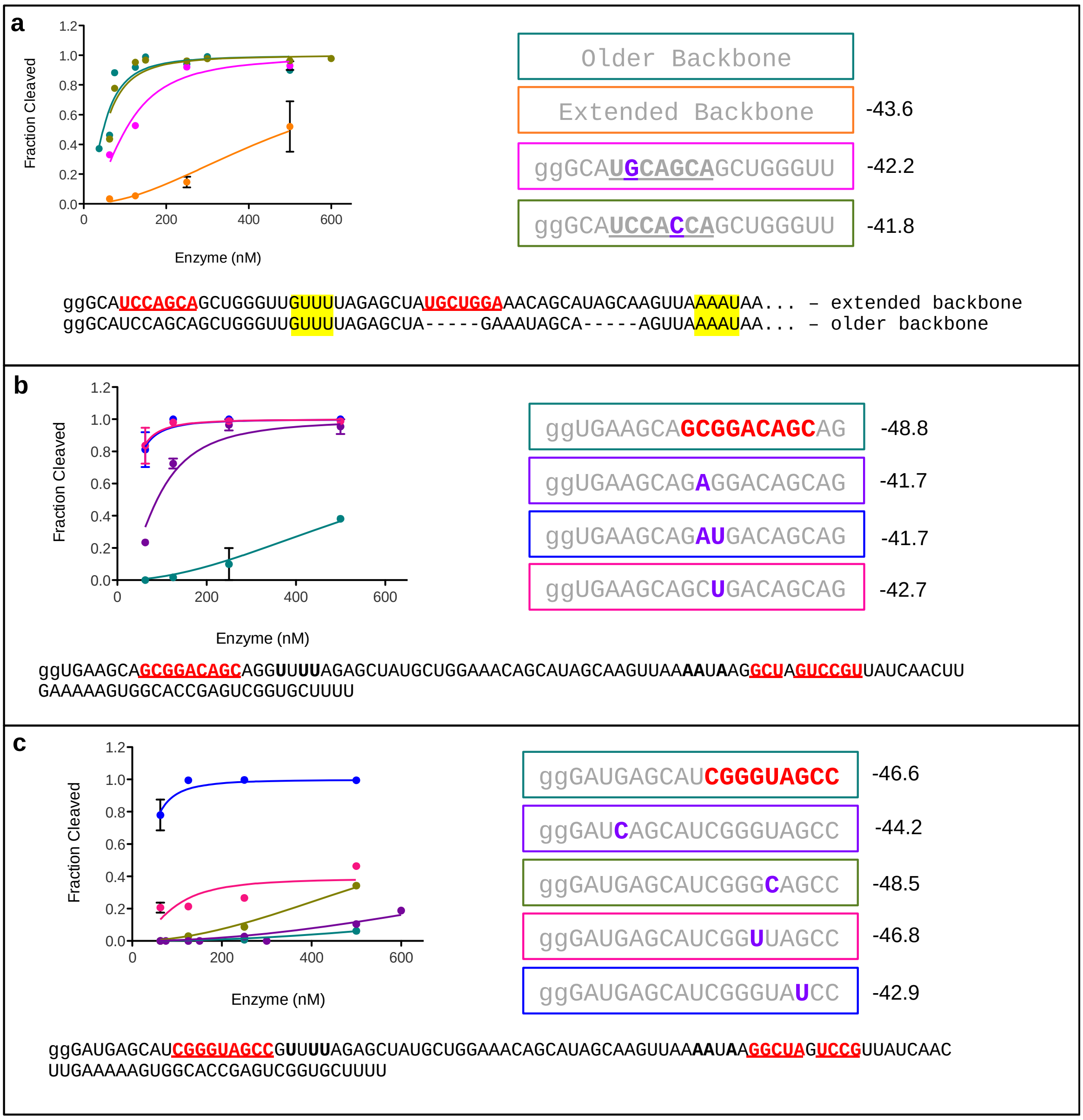

Supplementary Figure 13. Full cleavage data for gRNAs where interactions with the constant region of the gRNA backbone were broken. Predicted interactions between the gene-specific region of the gRNA and the gRNA backbone are shown in red and underlined. **a**) The gene-specific region was tested with two different published gRNA backbone sequences. The extended backbone was used in all work in this paper except in this one comparison case, and the edges of the backbone hairpin are highlighted in yellow, corresponding to the yellow hairpin 1 in Supplementary Fig. 14. This gRNA showed high cleavage activity in the context of the original backbone, but not the extended one, indicating that interactions were forming with part of the extension. Making single nucleotide substitutions in the gene-specific region, in the context of the previously inactive extended backbone, corrected the low activity. The Mfold free energy predictions for these substitutions do trend correctly, however they are not valid because the prediction does not identify this interaction between the gene-specific region and backbone, while the experimental evidence clearly supports it. **b** and **c**) Making single nucleotide substitutions in the gene-specific region for both of these gRNAs, both with interactions to the same predicted region of the backbone, resulted in increased activity comparable to substitutions in the backbone itself (Fig. 3f). Mfold free energy predictions with the whole backbone yielded structures and free energies that matched the expected interactions, for both **b** and **c**.

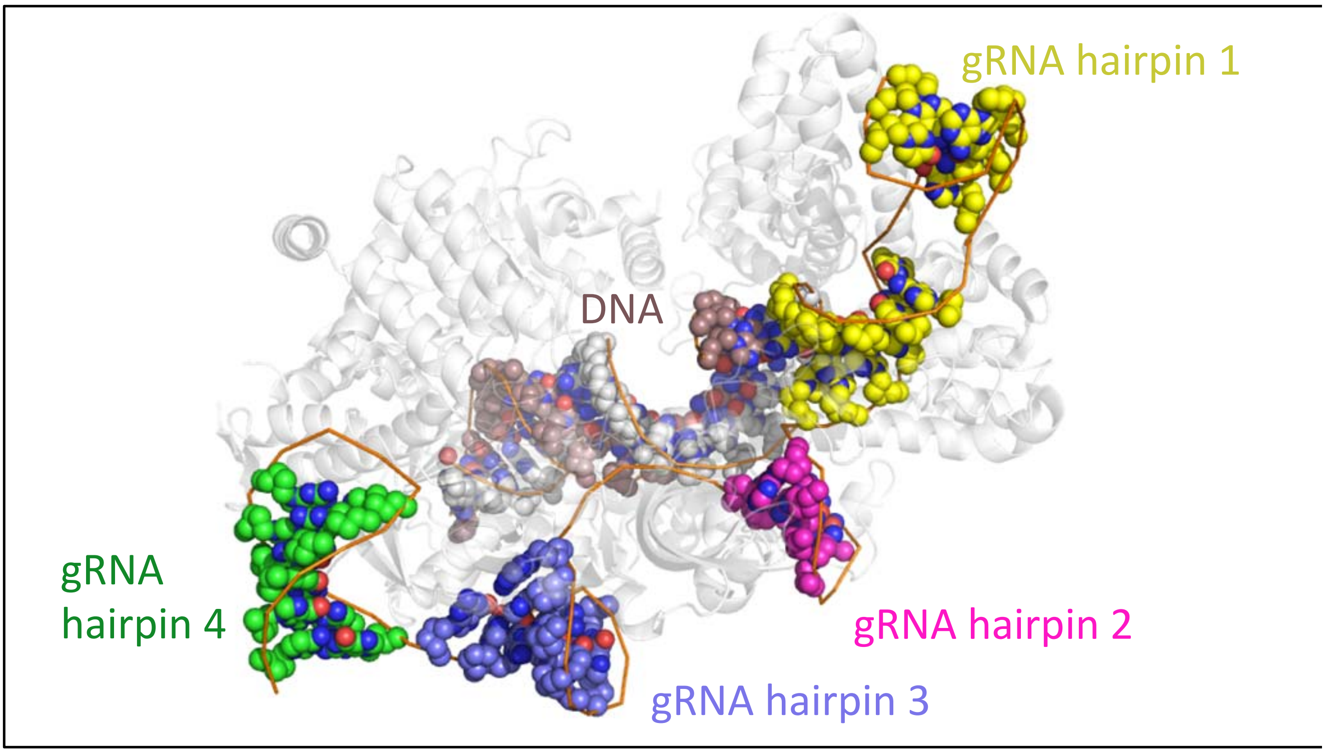

Supplementary Figure 14. Internal interactions in the gRNA backbone. The structure of the Cas9 complex with the gene-specific region of the gRNA shown white, the DNA in brown, and hairpins that occur within the gRNA backbone and stabilize the Cas9-gRNA complex in four different colors. The hairpin that is most susceptible to misfolding caused by interactions with the gene-specific region of the gRNA is hairpin 2. This hairpin has fewer base-pairs (4 compared to 6 and 7 for hairpins 3 and 4, respectively). The first region (hairpin 1) is the longest by total numbers of interactions, but it contains two region of strong interactions separated by a less stable region. This hairpin was also susceptible to misfolding when it was extended by ten base-pairs (Supplementary Fig. 13). This extension was predicted to stabilize the hairpin, but it is unclear whether it had the intended effect without further structural studies. All assays conducted in this work used the extended hairpin unless otherwise noted, as in Supplementary Fig. 13.

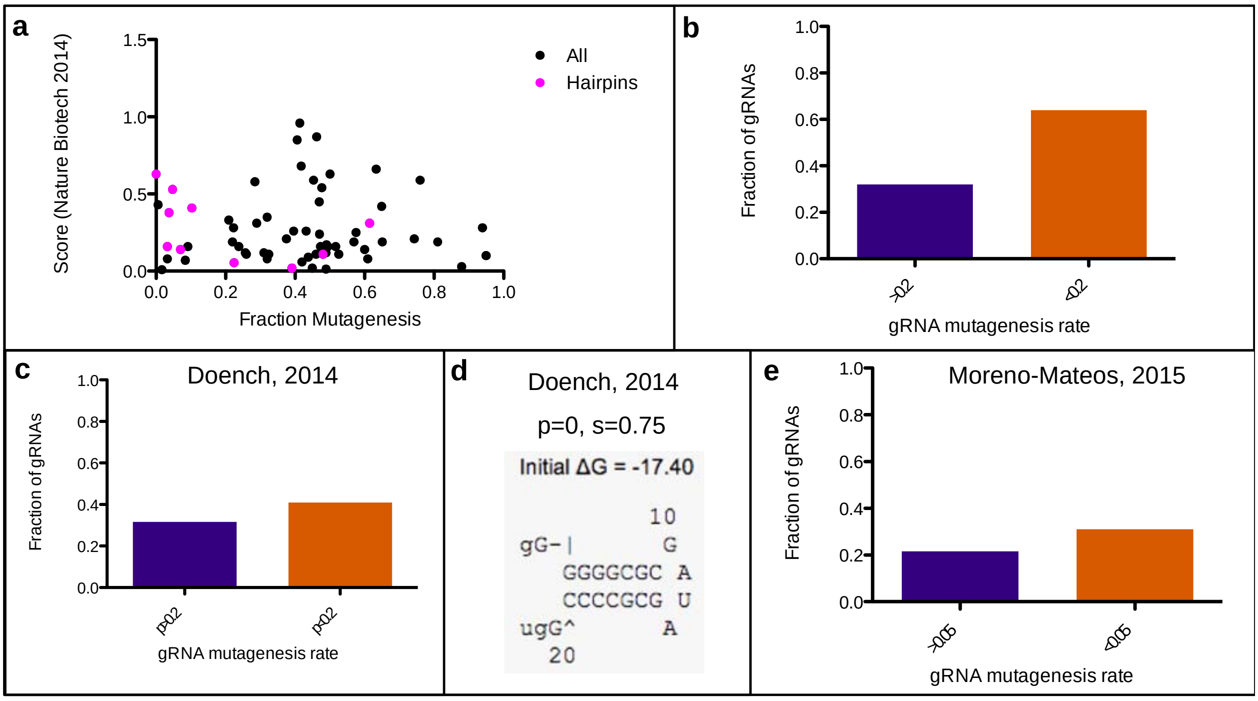

Supplementary Figure 15. Effect of hairpins in multiple datasets. a) Scoring of the gRNA activity of our dataset using the algorithm in Doench et al. (2014). A high score means that the gRNA should cleave better. We found that gRNAs with low cleavage likely due to hairpins are scored relatively well with this algorithm. **b**) Hairpins identified using our Python script in gRNas with low and high mutagenesis rates in our set of 67 carefully tested (made more than once and injections repeated) gRNAs. **c**) Hairpin prediction for the dataset of over 1,800 gRNAs from the Doench, et al. (2014) manuscript. The gRNAs from their lowest percentile activity group (<0.2) were compared with all other gRNAs (percentile>0.2). **d**) An example of a gRNA from the Doench, et al. (2014) dataset with a very low experimental cleavage (p=0), but a relatively high score (s=0.75). This mis-predicted gRNA contains an extremely strong hairpin (predicted with Mfold) within the gene-specific region and this interaction is almost certainly the reason for its low activity. **e**) Hairpin prediction for the dataset from the Moreno-Mateos, et al. (2015) manuscript. The gRNA mutagenesis rate for these gRNAs was collected from cleavage in zebrafish embryos using pools of gRNAs. For this set we compared gRNAs with a mutagenesis rate of less than 0.05 to all the rest of the gRNAs. We used a different criteria here compared to our own dataset because the tests of pooled gRNAs likely underestimate the mutagenesis induced by each gRNA due to limiting gRNA concentrations and potential competition effects.

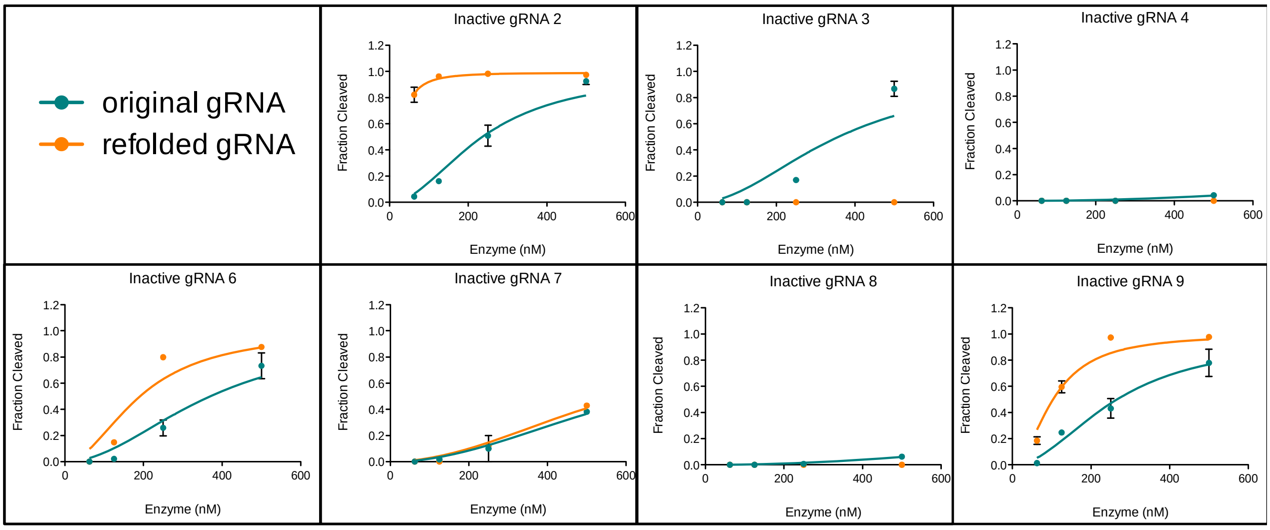

Supplementary Figure 16. Full cleavage data for gRNAs before and after refolding. Target site cleavage traces for gRNAs shown in Fig. 4. Heating and slowly cooling the gRNAs resulted in increased cleavage activity for some inactive gRNAs.

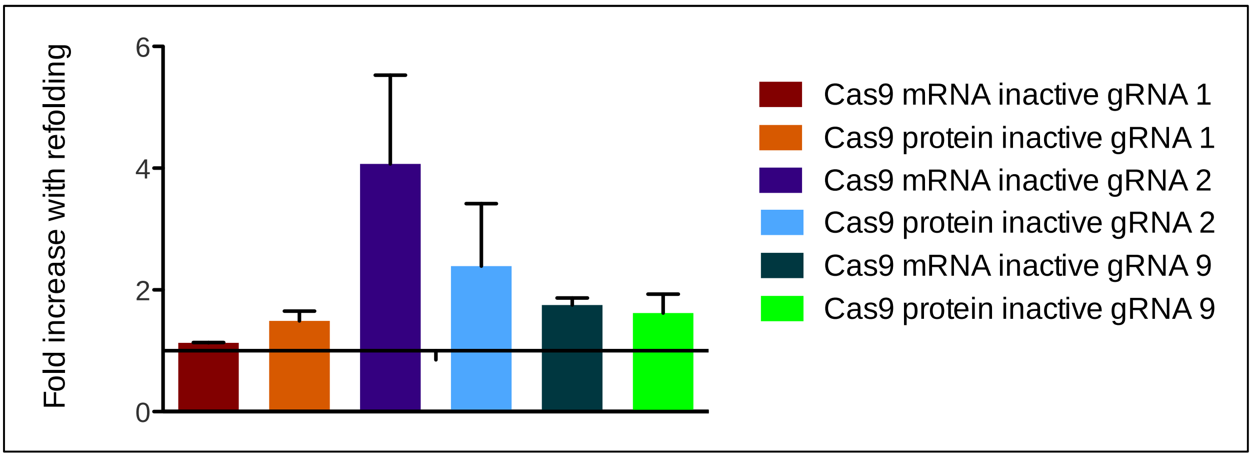

Supplementary Figure 17. Cleavage *in vivo* after refolding. Increase of *in vivo* cleavage for three gRNAs after refolding, both with Cas9 mRNA and protein.

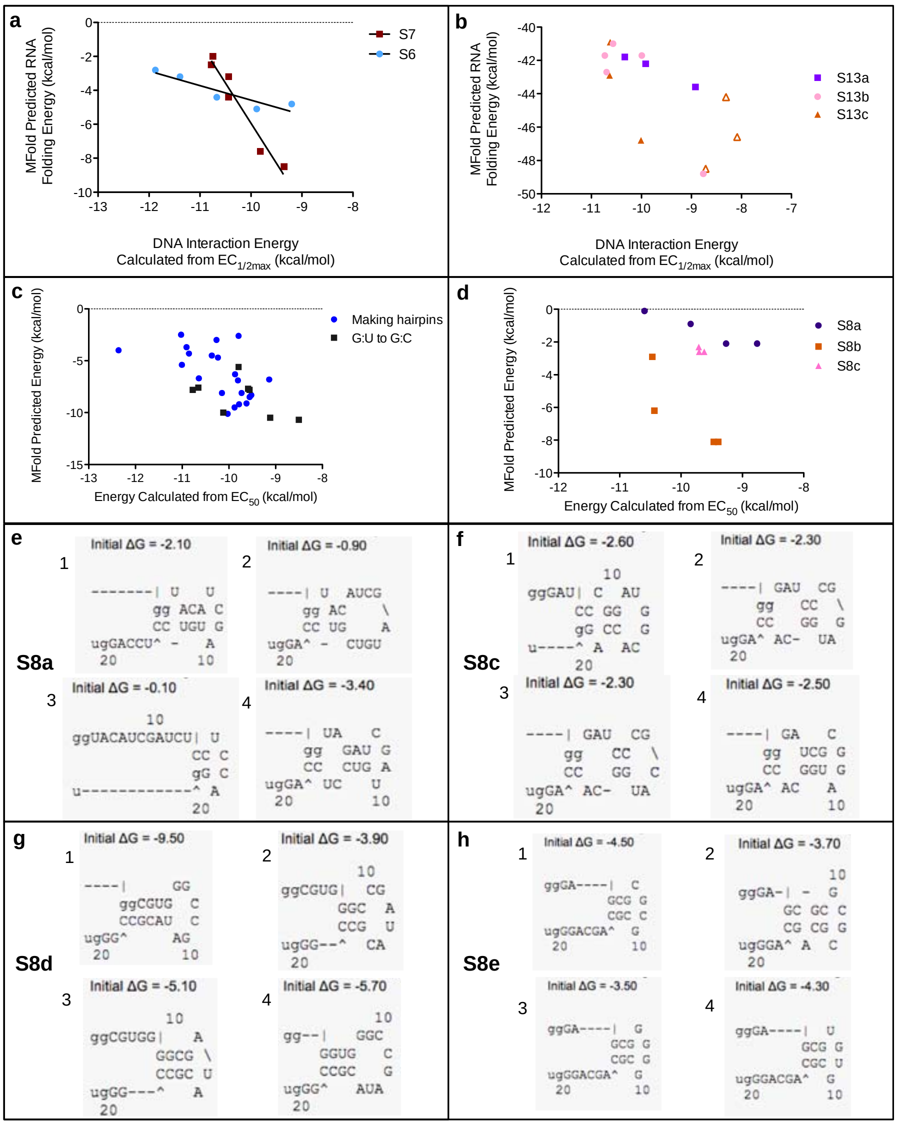

Supplementary Figure 18. Comparison of Mfold predicted free energies and experimental data. The experimentally-derived ΔG of Cas9 binding was estimated using the $EC_{1/2max}$ as a proxy for K_D. This assumption is valid if all Cas9-gRNA complexes have similar turnover rates and if their cleavage of the DNA is controlled by enzyme concentration. **a**) The correlation between the experimentally-derived free energy and Mfold free energy of gRNA folding for the sets of gRNAs shown in Supplementary Fig. 6 (S6) and 7 (S7). For both sets the predictions are accurate, R^2 = 0.85 for S6 set and 0.95 for S7, although the relative trends for each set differ from each other. **b**) Energies for breaking interactions between the gene-specific region and gRNA backbone shown in Supplementary Fig. 13. The S13a set displays a linear trend but, as discussed in the S13 legend, the values are not based on a correct structure. The S13b and S13c datasets match expectations reasonably well, especially when considering that several of the points (open triangles) are based on experimental $EC_{1/2max}$ values that are underestimated because the cleavage is so poor. **c**) Free energies for gRNA pairs where hairpins were increase and G:U pairings were substituted with G:Cs (Supplementary Fig. 10 and Supplementary Fig. 11). **d**) Free energies for sets of broken hairpins that are not implausible from Supplementary Fig. 8 (nothing in red text is shown here). **e-h**) Structures of Mfold-predicted interactions that correspond with predictions in Supplementary Fig. 8. **e**) Structure 1 is predicted for the wild-type sequence and gRNAs with low cleavage and high free energy break this sequence (2, 3), except for the high-cleaving gRNA with a low free energy prediction (4), that is likely not forming the predicted structure. **f**) As in **e**, structure 4 is predicted, but not likely forming for S8c. **g**) Examples of the putative hairpins for the wild-type sequence in S8d (1,2) and two low-cleaving mutant sequences (3,4) with potentially different detrimental hairpins. **h**) Putative interactions for the wild-type sequence in S8e (1,2), compared to alternative low-energy structures that are found for the high-cleaving gRNAs (3,4), but that are likely not forming. Structure 2 is compatible with the experimental data.

Supplementary Table 1. Sequences and genomic locations of gRNA targets shown in Fig. 1. Sequence details for (a) *in vivo* inactive gRNAs (Fig. 1c, Supplementary Fig. 3), (b) gRNAs neighboring inactive gRNAs 2-5 (neighbors of inactive gRNA 1 are shown in Fig. 1b), lettered sequentially from left to right as shown in the Fig. 1c and Supplementary Fig. 3 graphs, (c) gRNAs containing a putative CTCF motif (highlighted in cyan), numbered from left to right as shown in the Fig. 1e graph, and (d) gRNAs neighboring CTCF sites shown in the Fig. 1f graph. The ZV9 location is for the first base of the target site. The PAM and the first two bases, always converted to gg in the gRNA and thus with possible mismatches between the gRNA and target, are designated with lowercase letters.

	gRNA Target	ZV9 Genomic Location
In vivo inactive gRNA 1	tgCTCATCAATGTCTTCTGGagg	Chr24:32814788
In vivo inactive gRNA 2	gaCAGGCCCGCTCAGACACGtgg	Chr15:1787790
In vivo inactive gRNA 3 (CTCF gRNA 1)	ggTGAGAATTCTCCAGCAGCtgg	Chr11:28956077
In vivo inactive gRNA 4	ggATGTTGTCACTCGCTGACagg	Chr5:71787014
In vivo inactive gRNA 5	ggTCCTGCTCGTTGGATTTCtgg	Chr2:7359488
Neighbor gRNA 2a	acGTGGAAGAGTCTGAGACCcgg	Chr15:1787773
Neighbor gRNA 2b	acTCTTCCACGTGTCTGAGCggg	Chr15:1787784
Neighbor gRNA 2c	gtCATAGGCATGGATGGGACagg	Chr15:1787807
Neighbor gRNA 2d	gtTCTGTCATAGGCATGGATggg	Chr15:1787812
Neighbor gRNA 2e	gcCGGATGCAGGGCACAGGTtgg	Chr15:1787834
Neighbor gRNA 3a	ctCTTGTGGCAGGCTTGGATtgg	Chr11:28955994
Neighbor gRNA 3b	gaAGACACAAAACCCTCGGCtgg	Chr11:28956044
Neighbor gRNA 3c	tcGGTTGCTCAGCCAGCTGCtgg	Chr11:28956065
Neighbor gRNA 3d	ttTTTCCTTTACATTGGAGTcgg	Chr11:28956098
Neighbor gRNA 3e	gaGGTCAGTTCTAATGCACTtgg	Chr11:28956164
Neighbor gRNA 4a	tcTTCAGTTTGATGACAGTCtgg	Chr5:71786983
Neighbor gRNA 4b	gtCATCAAACTGAAGAGCGAtgg	Chr5:71786968
Neighbor gRNA 4c	ggAAGACATGTCAAAGGAGAcgg	Chr5:71786992
Neighbor gRNA 4d	tgACAGGGAAGACATGTCAAagg	Chr5:71786998
Neighbor gRNA 4e	gaATGCGAAGCTCCGAGAGCtgg	Chr5:71787035
Neighbor gRNA 4f	ggAGCTTCGCATTCGGCTGCcgg	Chr5:71787044
Neighbor gRNA 4g	gaTGTCTACAGTCACGCGTCtgg	Chr5:71787079
Neighbor gRNA 4h	caCAATGCTGTTTATGCTGAtgg	Chr5:71787104
Neighbor gRNA 5a	gcAGGGCAGCTGATACCTACtgg	Chr2:7359457
Neighbor gRNA 5b	gaTTTCTGGGTTTATCTGCAggg	Chr2:7359474
Neighbor gRNA 5c	gtCCACTTGGTCCTGCTCGTtgg	Chr2:7359496

CTCF gRNA 2	gcAGCTGTGCCTCCAGCAGCtgg	Chr2:34509728
CTCF gRNA 3	ttTTTCGGCTCCCCAGCTGCtgg	Chr1:43550681
CTCF gRNA 4	agGTTTTATCAGCCAGCTGCtgg	Chr7:41713732
CTCF gRNA 5	tgTAGGTATTCTCCAGCAGCtgg	Chr23:28164818
CTCF gRNA 6	ccAGTTTGAGCTCCAGCAGCtgg	Chr25:35737001
CTCF gRNA 7	caGCTGCTGGAGTGGGCCTGtgg	Chr14:32065319
CTCF gRNA 8	gaAGAGCATTCTCCAGCAGCcgg	Chr23:13282198
CTCF gRNA 9	gtCCGATGGGCTCCAGCAGCtgg	Chr18:2523989
CTCF gRNA 10	caCAAAAAGGCTCCAGCAGCtgg	Chr3:42224306
CTCF 2 Neighbor	agCCCGATGAAGGATGGCACcgg	Chr2:34509648
CTCF 6 Neighbor	taGAGGCAGCGGAGGAGCAGcgg	Chr25:35736964
CTCF 7 Neighbor	gcTCTGGCGAGGACAGAGGGagg	Chr14:32065253

Supplementary Table 2. Sequences of inactive gRNAs shown in Fig. 2 and Fig. 4.

	gRNA Sequence
Inactive gRNA 1	ggUACAUCGAUGUCCUCCAG
Inactive gRNA 2	ggACUCUGCGGUGAACCGCU
Inactive gRNA 3	ggACAGCGGGCGCUGUAAUC
Inactive gRNA 4	ggGCGUCUAGCGCCCGCGCG
Inactive gRNA 5	ggUGAAGCAGCGGACAGCAG
Inactive gRNA 6	ggUCCAGGACAUGGACCGGA
Inactive gRNA 7	ggGAUGAGCAUCGGGUAGCC
Inactive gRNA 8	ggGAUGAGCAUCGGGUAGCC
Inactive gRNA 9	ggCGUGGGCCGAUACGCCGG

Supplementary Table 3. Sequences of active gRNAs from Fig. 2 competition assays.

	gRNA Sequence
Active gRNA 1	ggAGGAUCACUGGUUUUGGA
Active gRNA 2	ggGAAGACAUUGAUGAGCAU
Active gRNA 3	ggAUAGACGGCAUCAAUCUU

Supplementary Methods

Sequence of random RNA used in competition experiments (was produced from a linearized plasmid with a T7 promoter):

GGCGAATTGGAGCTCCCGCGGTGCGGCCGCTCTAGAACTAGTGGATCCCCCGGGCTGCAGCCCAATGT
GGAATTCGCCCTTAAGGGCGAAGGGATGAGCATCGGTTCATCCGGGGGCTTCGGTTCAGGAGGCCAAG
GGGGATCCCGCATGGCACCAGTGGGGTAGAGGCGACCCTGCAGGAGGTTCCACAGTGGAT

CTCF PSSM

key	A	C	G	T
1	5	-1	5	5
2	5	5	5	-1
3	5	-1	5	5
4	5	-1	5	5
5	-1	5	5	5
6	5	5	-1	5
7	5	-1	5	5
8	-1	5	5	5
9	5	5	-1	5
5	5	-1	5	5
11	5	5	5	-1
12	5	5	-1	5
13	5	5	-1	5
14	5	-1	5	5
